# Machine learning-based technique for gain and resonance prediction of mid band 5G Yagi antenna

**DOI:** 10.1038/s41598-023-39730-1

**Published:** 2023-08-03

**Authors:** Md. Ashraful Haque, Md Afzalur Rahman, Samir Salem Al-Bawri, Zubaida Yusoff, Adiba Haque Sharker, Wazie M. Abdulkawi, Dipon Saha, Liton Chandra Paul, M. A. Zakariya

**Affiliations:** 1 Department of Electrical and Electronic Engineering, Universiti Teknologi PETRONAS, 32610 Seri Iskandar, Perak Malaysia; 2 Department of Electrical and Electronic Engineering, Daffodil International University, Birulia, Dhaka Bangladesh; 3 Space Science Centre, Climate Change Institute, Universiti Kebangsaan Malaysia (UKM), 43600 Bangi, Malaysia; 4 Department of Electronics and Communication Engineering, Faculty of Engineering and Petroleum, Hadhramout University, 50512 Al-Mukalla, Hadhramout Yemen; 5 Faculty of Engineering, Multimedia University, 63100 Cyberjaya, Selangor Malaysia; 6 Department of Electrical Engineering, College of Engineering in Wadi Addawasir, Prince Sattam Bin Abdulaziz University, Al-Kharj, Saudi Arabia; 7 Department of Electrical, Electronic and Communication Engineering, Pabna University of Science and Technology, Pabna, Bangladesh; 8 Smart Infrastructure Modelling and Monitoring (SIMM) Research Group Institute of Transportation and Infrastructure Universiti Teknologi PETRONAS, 32610 Bandar, Seri Iskandar, Perak Malaysia

**Keywords:** Electrical and electronic engineering, Aerospace engineering

## Abstract

In this study, we present our findings from investigating the use of a machine learning (ML) technique to improve the performance of Quasi-Yagi–Uda antennas operating in the n78 band for 5G applications. This research study investigates several techniques, such as simulation, measurement, and an RLC equivalent circuit model, to evaluate the performance of an antenna. In this investigation, the CST modelling tools are used to develop a high-gain, low-return-loss Yagi–Uda antenna for the 5G communication system. When considering the antenna’s operating frequency, its dimensions are ${0.642}\lambda _0\times {0.583}\lambda _0$. The antenna has an operating frequency of 3.5 GHz, a return loss of $-43.45$ dB, a bandwidth of 520 MHz, a maximum gain of 6.57 dB, and an efficiency of almost 97%. The impedance analysis tools in CST Studio’s simulation and circuit design tools in Agilent ADS software are used to derive the antenna’s equivalent circuit (RLC). We use supervised regression ML method to create an accurate prediction of the frequency and gain of the antenna. Machine learning models can be evaluated using a variety of measures, including variance score, R square, mean square error, mean absolute error, root mean square error, and mean squared logarithmic error. Among the nine ML models, the prediction result of Linear Regression is superior to other ML models for resonant frequency prediction, and Gaussian Process Regression shows an extraordinary performance for gain prediction. R-square and var score represents the accuracy of the prediction, which is close to 99% for both frequency and gain prediction. Considering these factors, the antenna can be deemed an excellent choice for the n78 band of a 5G communication system.

## Introduction

Nowadays, to address growing communication challenges in terms of size, bandwidth, and gain, the demand for newer microwave and millimetre-wave systems has increased. As a result, antennas are frequently used to suit the demands of satellite communication. Different satellite communication applications are available in different frequency ranges^1^. Investigators are constantly trying to improve the bandwidth and gain for antennas. In recent years, technology has grown very quickly, and both developed and developing nations now employ wireless communications at an extremely high level^2^. Recent decades have seen widespread adoption of numerous generations of wireless communication standards, such as 1G, 2G, 3 G, 4G, 5G, etc.^3–5^. The fifth generation of cellular technology (5G), which offers data speed in Gigabits/sec (Gbps), virtually eliminates the drawbacks of earlier technology. More so, 5G enables low-power IoT applications, which are expanding rapidly^6,7^. As the key frequency band for the rollout of 5G, the sub-6 GHz range (from 2 to 6 GHz) is expected to strike good stability between coverage and capacity, notably in the N77, N78, and N79 bands^8^.

Shintaro Uda and Hidetsugu Yagi were the inventors of the Yagi antenna, also known as the Yagi–Uda antenna. This antenna is directional and constructed with a dipole and a bunch of parasitic elements. The parasitic elements are one reflector set behind the dipole and more than one director set in front of the dipole element, which can improve radiation properties. It has directional radiation because it concentrates its signal in a single direction, making it less susceptible to interference from other transmitters^9^. There are many reasons for the Yagi Uda antenna’s widespread use, including its low price, substantial gain, and simple construction. While televisions were the primary users of this antenna in the early days after its invention, such devices now find usage in sectors as diverse as radar, radio frequency identification, satellite communications, and more^10^. In^11^, microstrip Yagi–Uda antennas were constructed with resonance frequencies close to 900 MHz, a substrate height of 1.575 mm, a characteristic impedance of 50 ohms, and a strip conductor thickness of 35 $\mu$ m using an RT Duroid 5880 material. Microstrip circuits are used to implement Yagi Uda antennas, allowing for the antennas to be small and discreet. A 5-element version of the Yagi–Uda antenna was developed in^12^ using the simulation software FEKO. The antenna’s centre frequency is 500 MHz, which can work with signals in the 450–550 MHz range, and the highest antenna gain is 6.7 dB. In^13^, numerous Quasi Yagi antennas are reviewed based on feeding methodologies. Some authors have reported gains of 14–17 dB for the Yagi–Uda antenna by increasing the number of directors. The Yagi–Uda antenna’s fundamental flaw is its narrow bandwidth^10,13^. A 3-D full wave electromagnetic simulations of a ground penetrating radar (GPR) is used for artificial-intelligence-based buried item characterization is represented in^14^. This work developed a fast and accurate data-driven surrogate modeling approach for buried objects characterization, a computationally efficient surrogate model construction method using small training datasets, and a novel deep learning method, time-frequency regression model (TFRM), that uses raw signal without pre-processing to achieve competitive estimation performance. The given method outperforms multilayer perceptron (MLP), Gaussian process (GP), support vector regression machine (SVRM), and convolutional neural network (CNN) regression. Authors are stated in^15^, frequency-reconfigurable antennas have their own generalizable surrogate modeling approach. The technique postprocesses CAD simulation discrete data to a surrogate model. Afterwards, a reconfigurable UWB antenna with a tunable notch band shows that surrogate modeling is practical, effective, and precise. The proposed surrogate model is a good contender for a cognitive radio system’s reconfigurable antenna-signal processing interface standard. Miniaturized microwave components are generally designed using full-wave electromagnetic (EM) simulations^16^. Surrogate-assisted procedures use rapid data-driven metamodels to replace costly EM simulations. Verification studies for three microstrip components show that the suggested approach outperforms performance-driven approaches and standard modeling processes in surrogate fabrication accuracy and computing cost. In^17^, authors are discussed about surrogate-assisted microwave filter designs utilizing different design objective functions. Surrogate modeling (machine learning) and advanced optimization algorithms are examined for filter design. Three basic filter design methods are: Smart data sampling, advanced surrogate modeling, and advanced optimization frameworks. They must be customized or blended to match microwave filter parameters for success and stability. Finally, emerging filter design applications and trends are examined. The researcher used surrogate modeling to design and optimize MIMO antennas in^18^. Microwave Studio and MATLAB numerical analyzer automatically optimize. Shallow neural network optimization is used to identify the best TARC, S11, and S12 solutions. A 3.1–10.6 GHz ultra-wideband MIMO antenna is constructed and optimized to test the suggested approach. Antennas are difficult to design and maintain without the use of machine learning technologies. Without machine learning, antenna design accelerates too slowly. Without ML, it’s hard to keep errors low and productivity high. Not having the helping Hand for ML Simulation reduction while maintaining work feasibility and antenna behavior calculation is a challenging task^19^. Machine learning replaces trial-and-error in metamaterial simulations by predicting design parameters using one or more properly designed machine learning models. Two things affect prediction accuracy. primarily dataset size. Furthermore, the training machine learning model^20^. An antenna-derived material ensemble approach estimates antenna bandwidth and gain in^21^. This paper compares the presented method to SVM, Random Forest, K-Neighbors Regressor, and Decision Tree Regressor. The adaptive dynamic polar rose guided whale optimization technique optimizes ensemble model features. The suggested model predicted antenna bandwidth and gain efficiencies better than the others in a regression study. Based on antenna specifications, machine learning techniques may forecast the reflection coefficient (S11). Thus, it can prevent the trial-and-error optimization loop. This research^22^, used Decision Tree, Random Forest, XGBoost Regression, KNN, and ANN algorithms. Since the simulation dataset is nonlinear, these algorithms were chosen to perform regression for nonlinear data. After antenna simulation using HFSS, this research obtains the L-shaped slot’s resonance frequency, length, width, and thickness. Different ML algorithms predict values. The prediction accuracy is measured by the R-square score and Mean Squared Error (MSE) for the simulated and predicted reflectance coefficients (S11). A Yagi-Uda antenna using an artificial neural network (ANN) to forecast antenna gain and training time is proposed in^23^. In^23^, only MSE was used as a prediction accuracy metric, while MAE, MSLE, RMSLE, MAPE, RMSE, R-Square, and Var scores were ignored. Furthermore, the suggested ANN model’s prediction results were not compared to those of other current ML models. In another study^24^, the authors probed one of the IoT’s key forms of communication, ambient backscattering, and suggested a machine learning-based antenna design strategy for physical layer protection. To determine the degree of inaccuracy in this study, the researchers did not calculate the percentage of error expressed as MSE, MAE, or RMSE. Furthermore, the variance score has not been quantified in the majority of the previous papers on ML-based antenna design.

In this paper, the proposed antenna has a suitable realized gain (6.57 dB) and bandwidth (520 MHz), and the antenna size ($0.642\lambda _0 \times 0.583\lambda _0$) is compact when compared with the Yagi–Uda antenna. The impedance response of a two-layer structure consisting of a single material was predicted. Therefore, a brand-new type of predictive model based on electrical equivalent circuits was created. Realizing an antenna’s desired performance levels in 3D electromagnetic simulation programs like CST, HFSS, FEKO, and ADS is a complex and time-consuming endeavor. In this research, nine regression ML models such as linear regression (LR), random forest regression (RFR), decision tree regression (DTR), lasso regression, ridge regression (RR), extreme gradient boosting (XGB) regression, Bayesian linear regression (BLR), Gaussian process regression (GPR) and Support Vector Regression Machine (SVRM) are used to predict the operating frequency and gain of the proposed antenna.

The CST MWS simulation software is used to design and optimize the antenna’s performance. Furthermore, the exact same antenna was remodeled in measurement to validate the performance result obtained from the simulation. The Advance Design System (ADS) circuit simulation tool is used to validate the return loss level and bandwidth by using the R-L-C equivalent circuit. Using the CST electromagnetic (EM) modelling tool, a novel approach has recently been explored predicting the frequency and gain with numerous unsupervised regression methods.

## Design of proposed antenna

**Figure 1:**
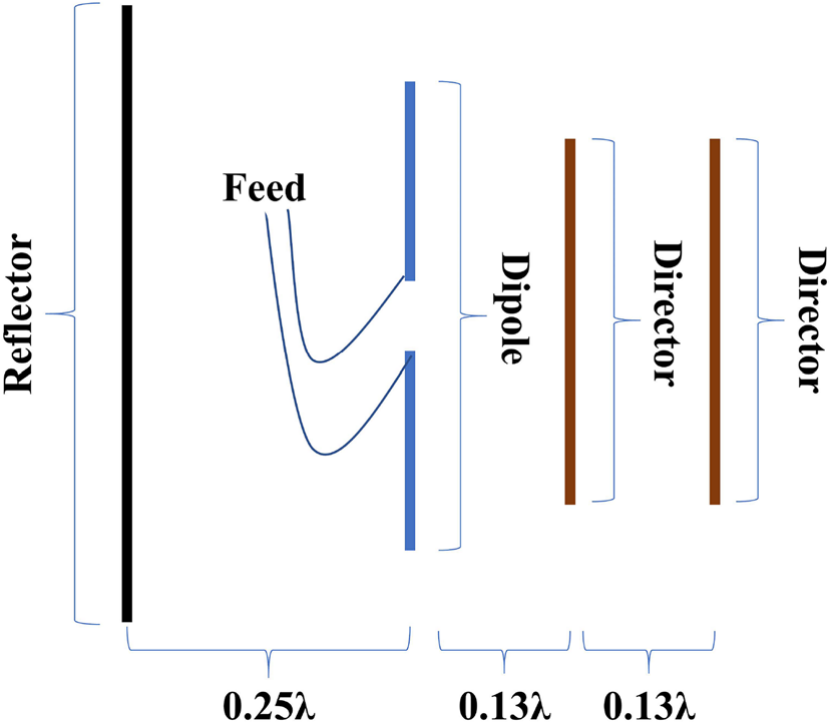
Structure of Yagi–Uda Antenna.

To model the performance of the proposed 5G antenna, employed the CST MW package from Computer Simulation Technology. The basic structure of the Yagi–Uda antenna is presented in Fig. 1.

It is often found that the reflector comes after the other two elements. Its typical length is 5% greater than that of the dipole element. The dipole element’s length is equal to half its wavelength. Notably, the spacing between the dipole element and the reflector can be between 0.1$\lambda$ and 0.25$\lambda$. Directors are placed at a length of 5% less than the dipole element. The spacing between the dipole element and each director is 0.13$\lambda$^25,26^. The value of $\lambda$, and the initial value of length of parasitic elements along with driven element and spacing between two elements can be calculated by using the following Equations (1, 2, 3, 4, 5, 6, 7)^25,26^:

1 $$\begin{aligned}&\textstyle \lambda = \frac{c}{f} \end{aligned}$$

2 $$\begin{aligned}&\text {Length of Driven or Dipole element} \textstyle = \frac{1}{2} \times \lambda \end{aligned}$$

3 $$\begin{aligned}&\text {Length of Reflector} = 0.55\times \lambda \end{aligned}$$

4 $$\begin{aligned}&\text {Length of Directors} = 0.45\times \lambda \end{aligned}$$

5 $$\begin{aligned}&\text {Spacing between Reflector and Dipole} = 0.25\times \lambda \end{aligned}$$

6 $$\begin{aligned}&\text {Spacing between Dipole and Director 1} = 0.13\times \lambda \end{aligned}$$

7 $$\begin{aligned}&\text {Spacing between Dipole and Director 2} = 0.26\times \lambda \end{aligned}$$

where *c* = Speed of light, *f* = Resonant Frequency, $\lambda$ = Wavelength

**Figure 2:**
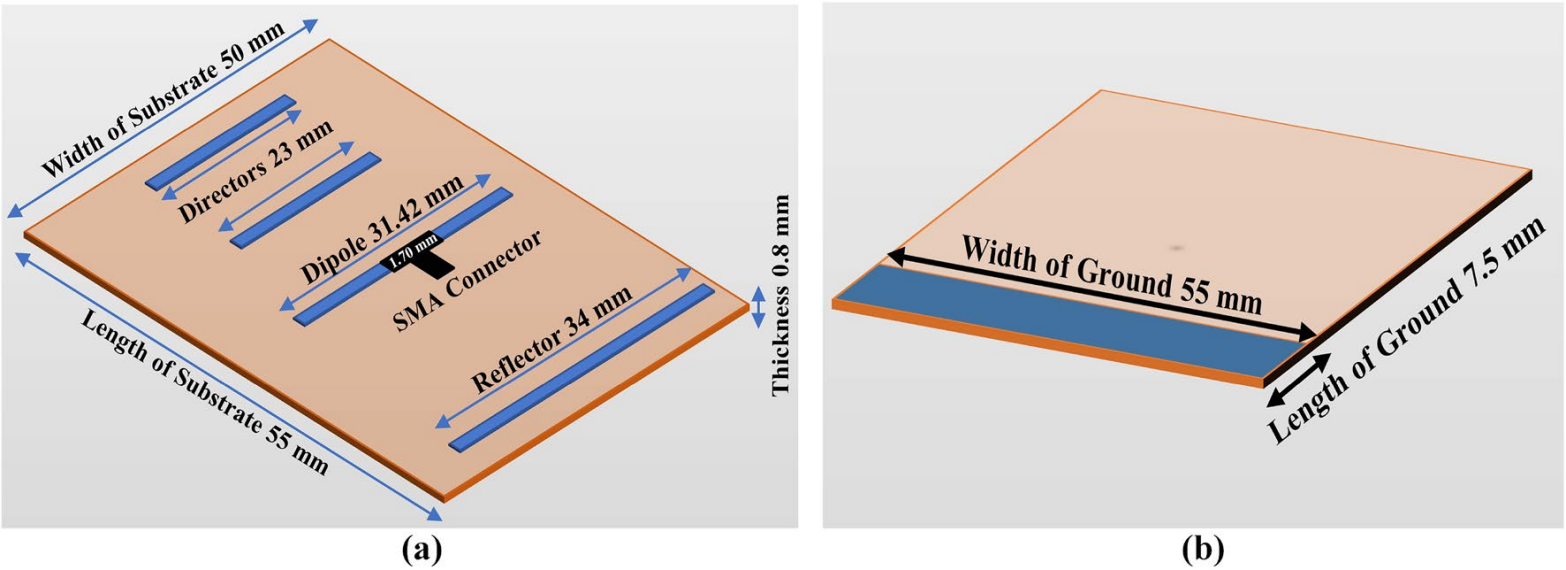
Dimensional (**a**) front and (**b**) back view.

FR-4 (lossy) substrate is used to design and simulate the antenna. The antenna’s total size is $0.642\lambda \times 0.583\lambda \times 0.009\lambda$. The thickness of the ground plane is 0.035 mm. The length of the reflector is 34 mm. The length of the dipole is 30.87 mm, and the length of the directors is 23 mm. The width of these elements is 1 mm. The length of the feed is 1.70 mm, and used a discrete port for simulating the antenna with a 50-ohm impedance. The front and back views are shown in Fig. 2.

**Table 1:** Performance comparisons with the recent state of the art.

Parameter	Ref.^27^	Ref.^28^	Ref.^29^	Ref.^30^	Ref.^31^	Ref.^32^	Ref.^33^	Proposed work
Technique	Quasi Yagi (2 port)	Finite integration technique	Vehicular	WLAN & 5G	Split-ring resonator	Dielectric resonator antenna	Cross-ring slot with DR truncation	Discrete port Microstrip Yagi
Oparating frequency (GHz)	3.6	3.8 and 5.2	2.4	4.28	2.45	3.72	3.25	3.5
Return loss (dB)	–29	–32	–25	–44.8	–27	–35	–35	–43.45
Bandwidth (%)	8	2.2–8	2.35–2.45	4–6	3.95	17.47	18.7	14.77
Peak gain (dB)	4.3	5	5.7	3	6.73	4.27	4.83	6.57
Radiation efficiency (%)	73	96	–	80	70	80	–	97
ML analysis	No	No	No	Yes	No	No	No	Yes
Size ($\hbox {W}\times \hbox {L}$)	0.47$\lambda _0 \times 0.93\lambda _0$	0.7$\lambda _0 \times 0.47\lambda _0$	0.8$\lambda _0 \times 0.8\lambda _0$	0.48$\lambda _0 \times 0.64\lambda _0$	0.593$\lambda _0 \times 0.48\lambda _0$	0.29$\lambda _0 \times 0.19\lambda _0$	0.5$\lambda _0 \times 0.5\lambda _0$	0.642$\lambda _0 \times 0.583\lambda _0$
Substrate Material	FR4 ($\varepsilon$=4)	FR4	FR4	PET	FR4 ($\varepsilon$=4.3)	FR4 ($\varepsilon$=4.4)	FR4 ($\varepsilon$=4.4)	FR4 ($\varepsilon$=4.3)

## Result analysis of the proposed antenna

The simulated and measured results of the proposed Yagi–Uda antenna are discussed in this section. The simulated S11 using CST is also compared with the result obtained from the ADS. Different machine learning algorithms are discussed in brief to predict the resonance frequency and gain of the proposed antenna. In Table 1. the comparison of performance with recently published work is presented.

### Perametric analysis

The impact of the structure’s primary parameters is illustrated in the following sections to help the reader comprehend it better.

#### Impact of dipole length

**Figure 3:**
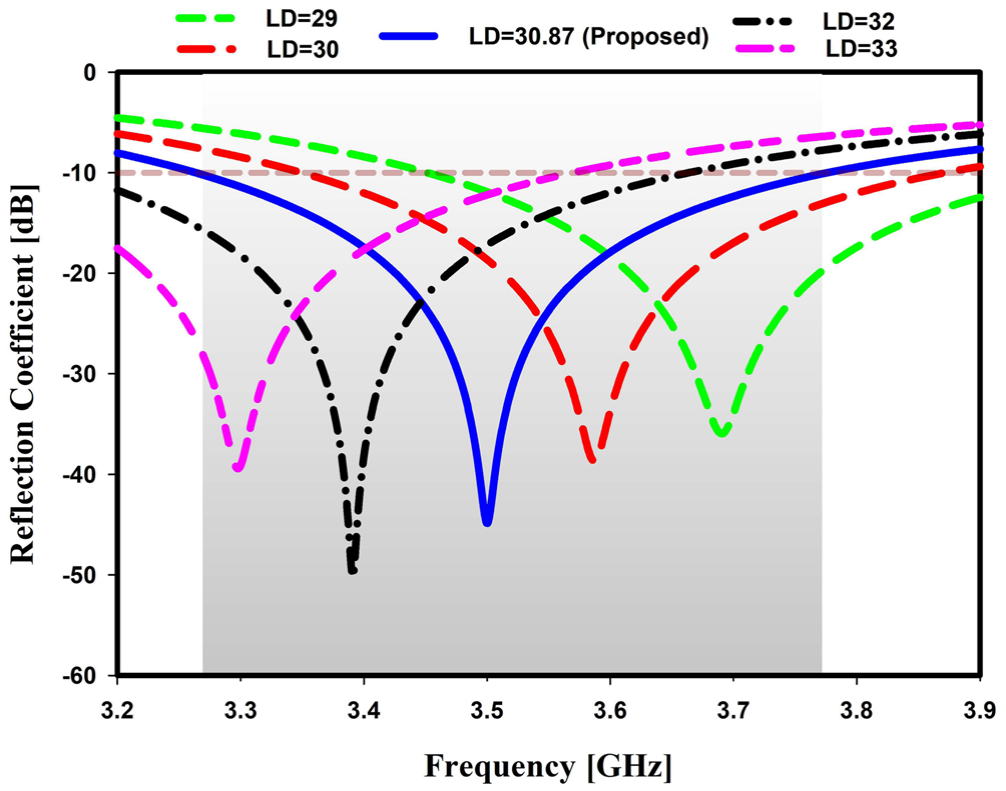
Simulated reflection coefficient for different length of dipole.

The dipole element of a Yagi antenna is frequently considered the essential portion of the antenna since it connects the antenna to its power source and serves as the feed. In this study, it has been noticed that as the length of the dipole increases, the return loss also increases and starts to decrease at a specific length. For this design, the particular length is 33 mm. Moreover, the resonance frequency also moved to the left when the length increased. The desired frequency of this study was 3.5 GHz, which was found at a length of 30.87 mm, presented in Fig. 3.

**Figure 4:**
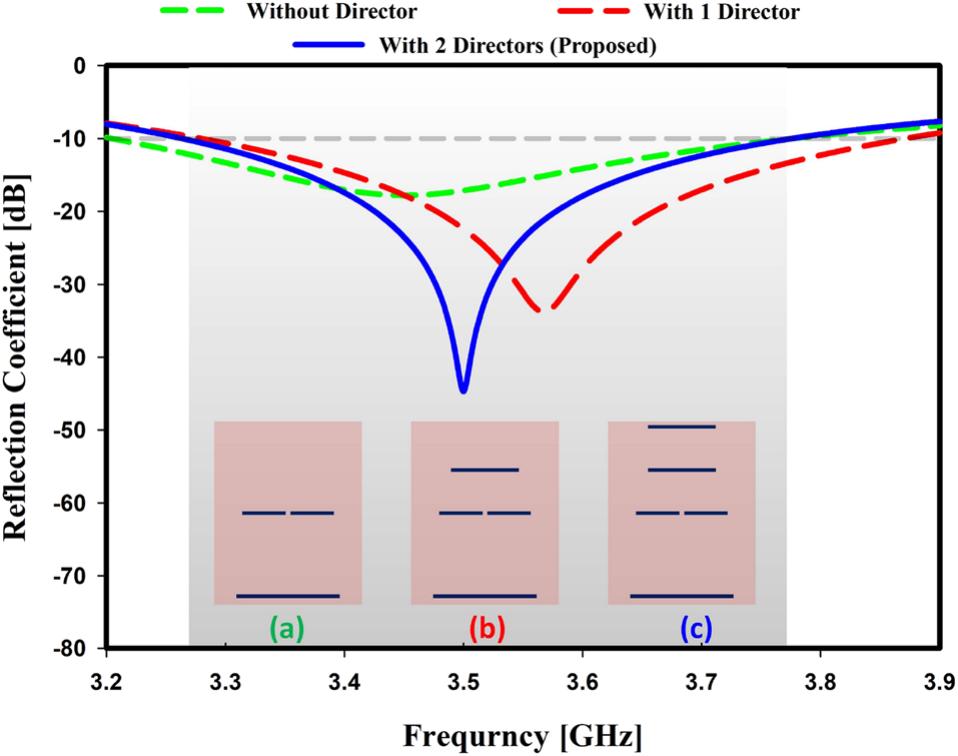
Simulated reflection coefficient for director.

**Figure 5:**
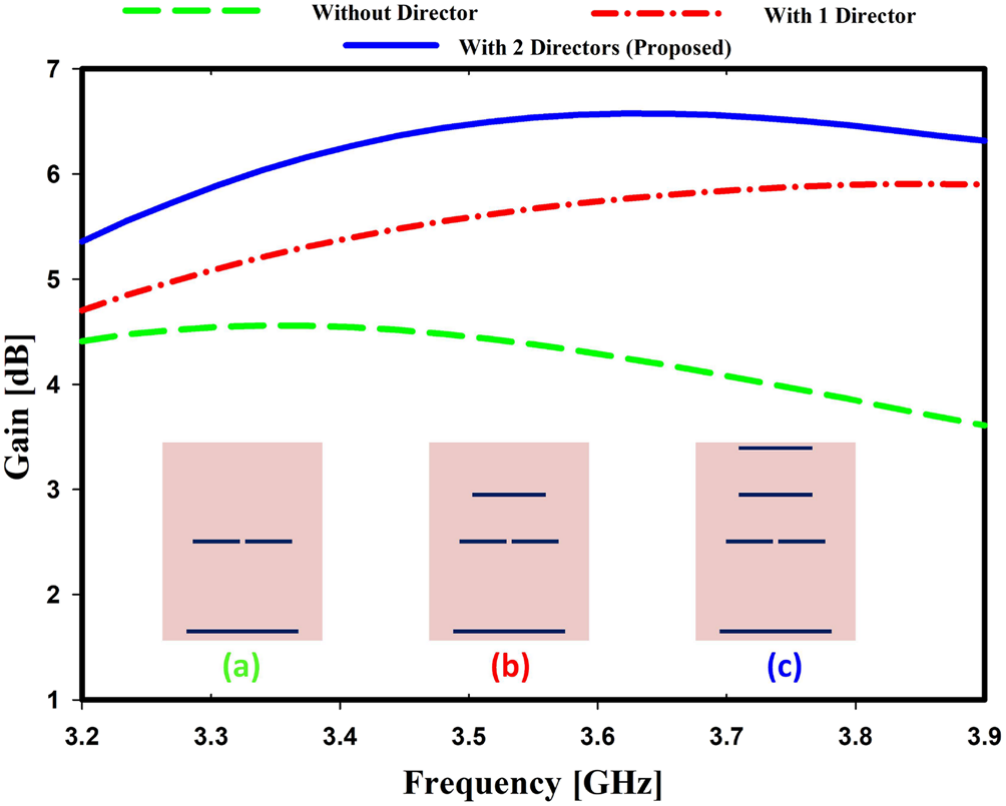
Simulated gain for different directors.

#### Impact of directors

The component on the right side of the Yagi antenna, known as the director, is in charge of concentrating the radiated power along the director components because of its capacitive nature^34^. Because of its radiative nature, it is also known as a parasitic element. There are used two directors in this study. The effect of directors on resonance frequency and return loss level is shown in Fig. 4 with and without directors. The suitable curve of the design was found when it was simulated with two directors. The level of return loss is less evident in the absence of directors. The resonance frequency is cleared with one director but not the desired one. Moreover, it is discovered that the return loss level is increased when directors are increased. An antenna’s gain increases when more directors are added after the dipole^35^. The director has a significant impact on the antenna’s gain, as shown in Fig. 5. Without directors, the gain is 4.45 dB at the resonance frequency, and it is 5.5 dB with one director and 6.57 dB with two directors.

### Current distribution

**Figure 6:**
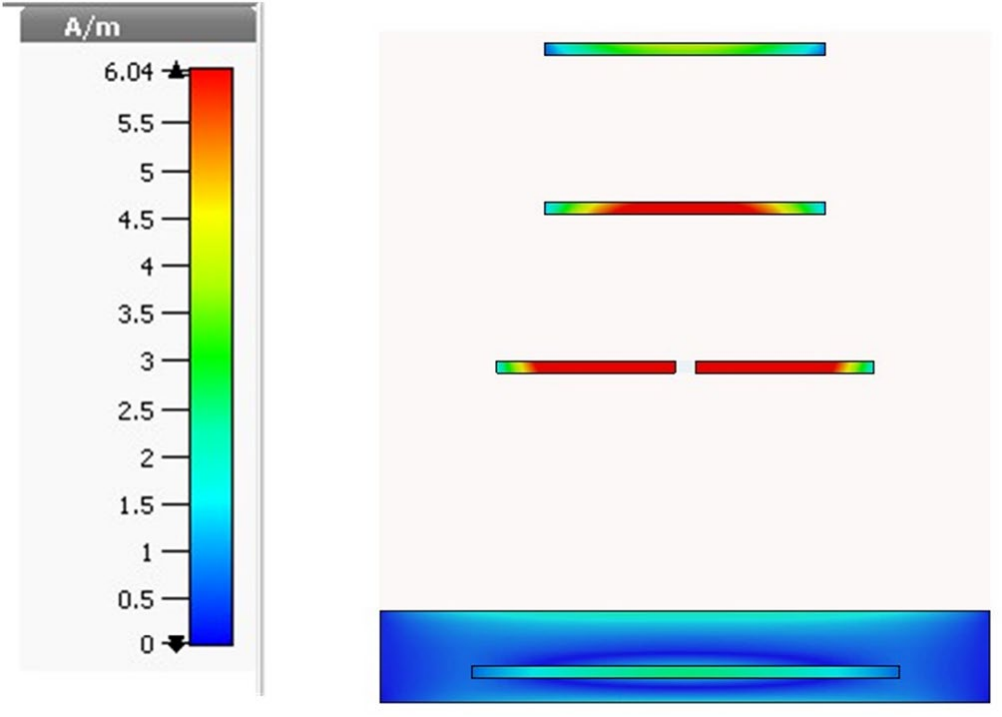
Current distribution at frequency 3.5 GHz.

At 3.5 GHz, a representation of the current distribution may be found in Fig. 6. At the center of the dipole, the current is at its maximum of 37.58 A/m before it is distributed to the first parasitic element. The intensity of the surface current is reflected in the color, which serves as a visual representation of the concept. At a frequency of 3.5 GHz, it is possible to detect a current that is traveling down the surface of the item.

### Simulation and measurement

**Figure 7:**
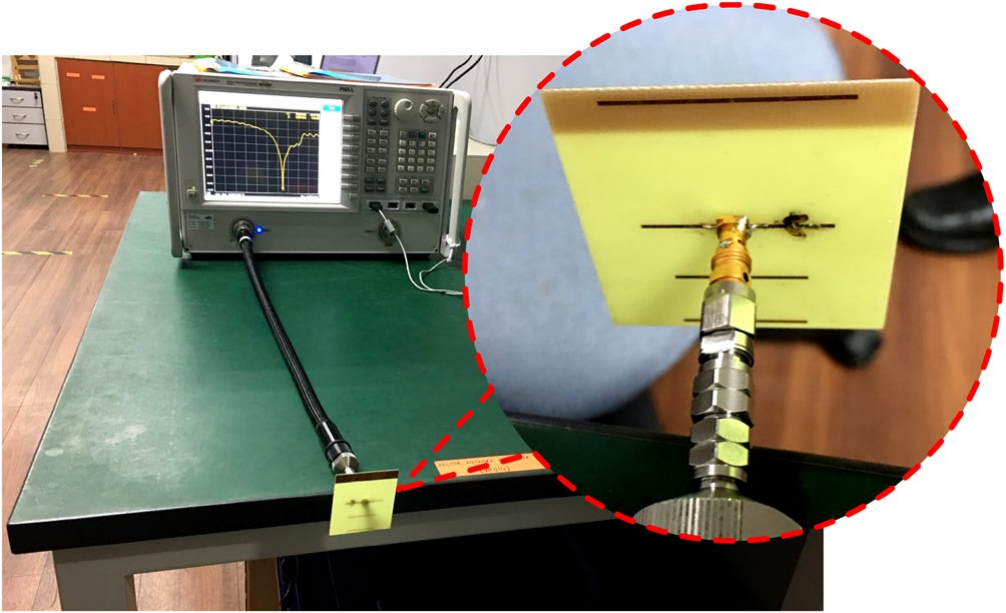
Return loss measurement using vector network analyzer.

**Figure 8:**
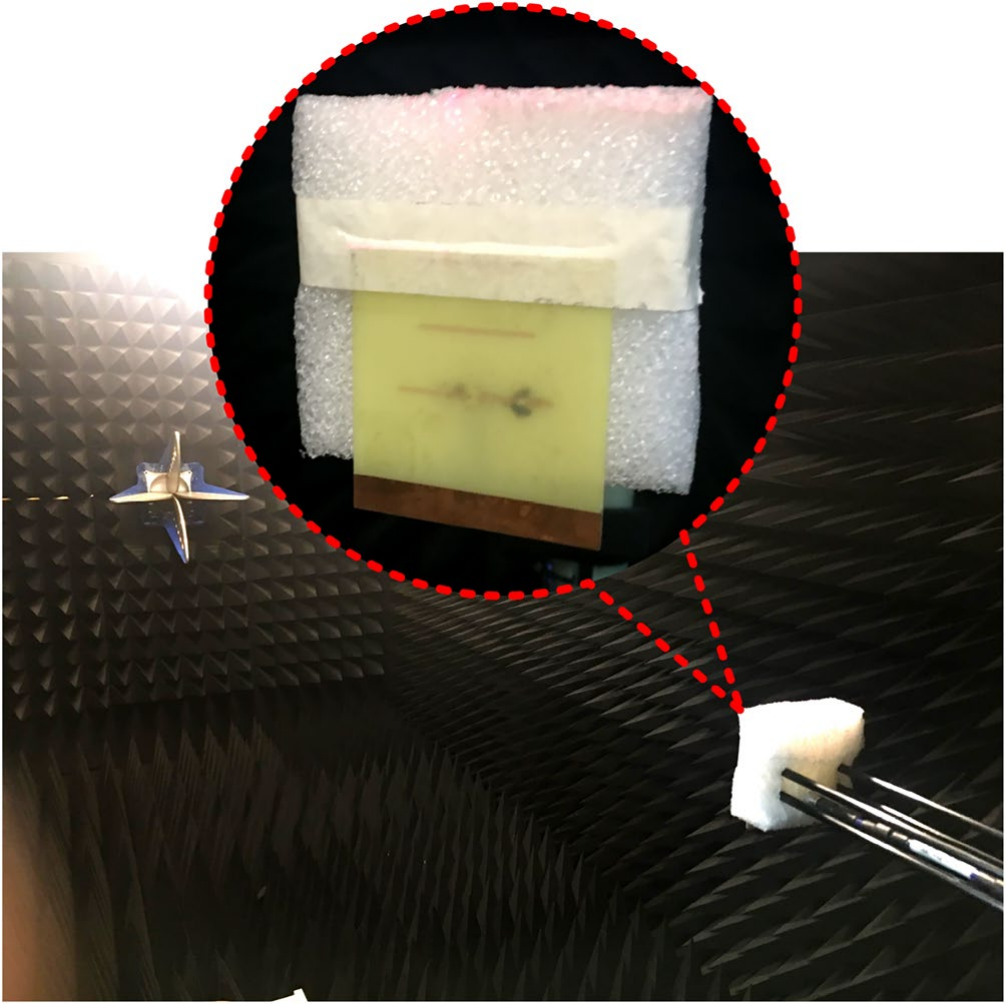
Radiation characteristics measurement within anechoic chamber.

A vector network analyzer (VNA), as shown in Fig. 7, is used to test the port qualities, while an anechoic chamber is used to examine the radiation properties shown in Fig. 8.

#### Return loss

The strength of a signal that is reflected from an antenna and travels back to the transmitter is known as the return loss (S1,1). A higher return loss indicates that the antenna can transmit more RF energy. Greater bandwidth is a necessary condition for 5G communications since it enables faster communication and data transfer^36^. For optimal performance, the return loss must be less than $-10$ dB, which is expressed as a decibel (dB)^37^.

**Figure 9:**
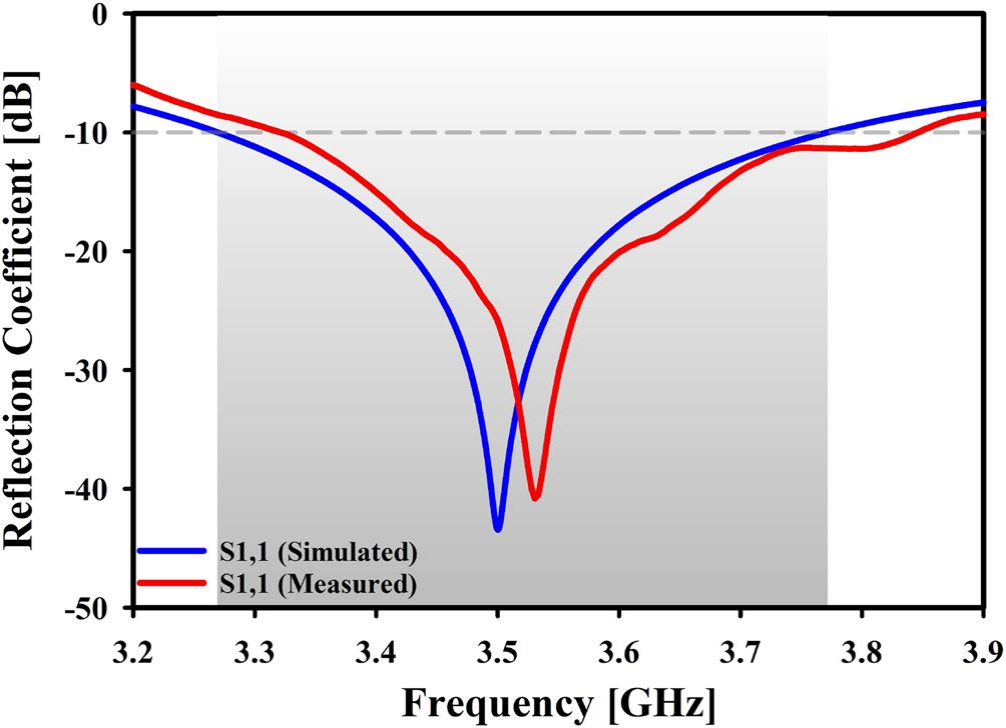
Simulated and measured reflection coefficient.

It can be seen that the observed resonance frequency is extremely close to the simulated one (Simulated: 3.50 GHz and Measured: 3.53 GHz). Approximately -43.45 dB (when simulated) and -40.81 dB (when measured) is the reflection coefficient at the resonant point as depicted in Fig. 9.

#### Gain and efficiency

When determining an antenna’s effectiveness, it is crucial to consider its gain and directivity. Gain quantifies how much energy is transferred to the primary beam, whereas directivity evaluates how much power is focused in a single direction^38^.

An antenna’s effectiveness is measured in part by its Gain and Directivity^39^. Efficiency calculated 96.76% by using the equation (8).

8 $$\begin{aligned} Efficiency=\frac{Gain}{Directivity}\times 100\% \end{aligned}$$

**Figure 10:**
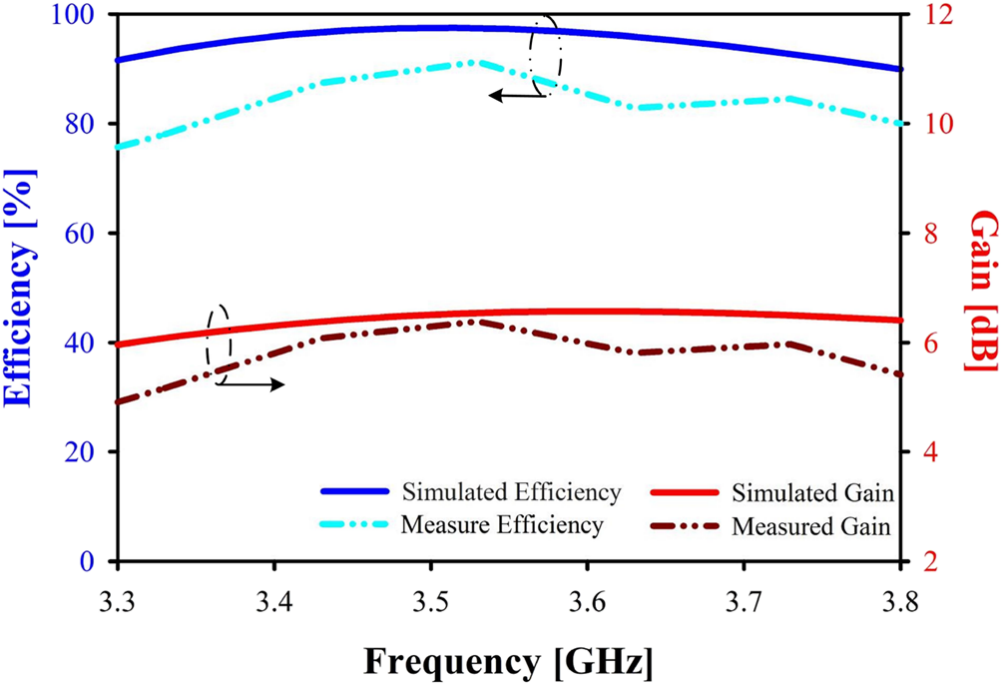
Simulated and measured gain & efficiency of proposed antenna.

Maximum gain values (simulated 6.57 dB) over the operating frequency band are displayed in Fig. 10, demonstrating the antenna’s suitability for the n78 5G band. In an anechoic chamber, the prototype’s peak gain was measured to be 6.39 dB. In addition, the range of simulated efficiencies and measured efficiencies, as shown in Fig. 10, varies from 84 to 97% for simulated efficiencies and 75–93% for measured efficiencies.

**Figure 11:**
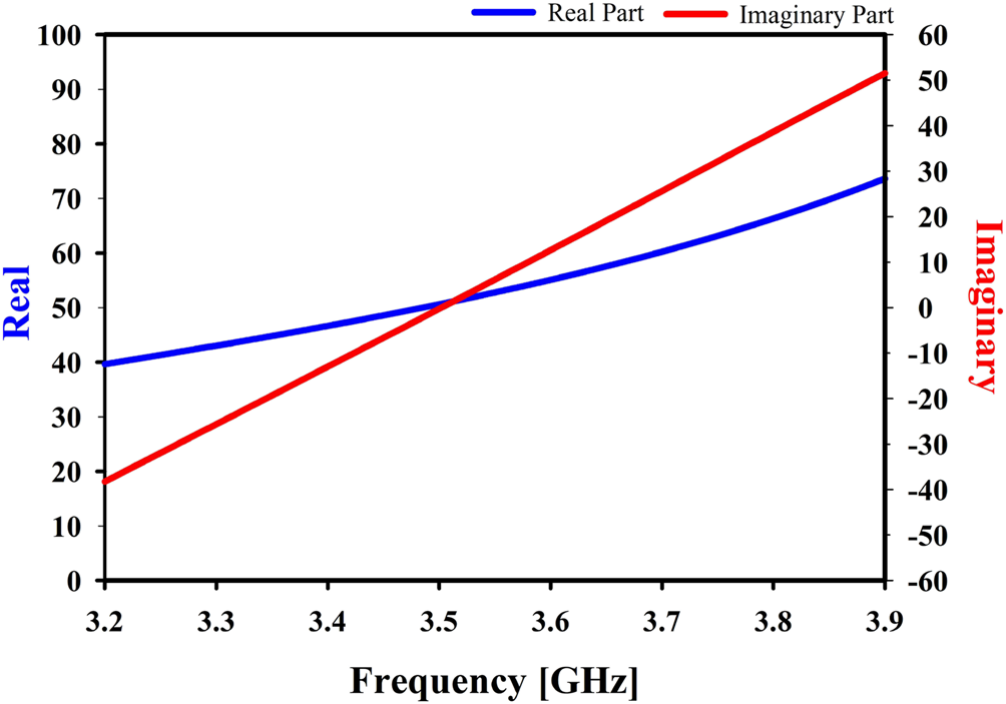
Z-parameter of the studied antenna.

The Z-matrix has highlighted yet another important essential impedance feature of the proposed Yagi antenna, as seen in Fig. 11. According to this figure, the real component of the Z-parameter is close to 50 ohm, whereas the imaginary component of the Z-parameter is close to 0 when the frequency is 3.5 GHz.

#### Radiation pattern (2D)

**Figure 12:**
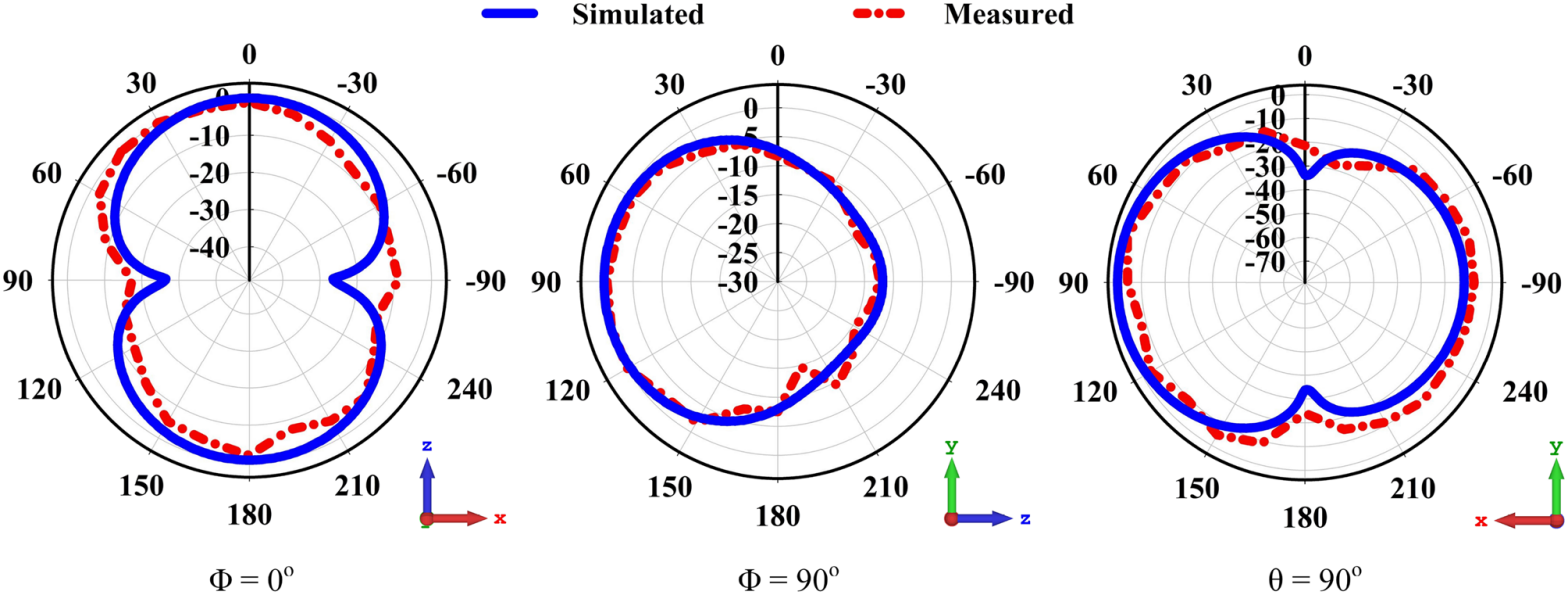
Simulated and measured 2D radiation pattern of proposed antenna at 3.5 GHz for $\Phi = 0^\circ$, $\Phi = 90^\circ$, $\theta = 90^\circ$.

The simulated and measured 2D radiation patterns for the frequency of 3.5 GHz are shown in Fig. 12. Since theta ($\theta$) and phi ($\Phi$) are circular coordinates, they can be used to describe the orientation of the radiation pattern in relation to the Cartesian axes; for example, if is approximately constant 0, then the region from 0$^\circ$ to 360$^\circ$ is the XZ cut, which is also known as the E-plane. Simulated and measured 2D radiation patterns are projected into the E-plane along the XZ ($\Phi$ = 0$^\circ$) & YZ ($\Phi$ = 90$^\circ$) axes the H-Plane along the xy direction at $\theta$ = 90$^\circ$. Extensive testing of the far-field properties reveals superior directional behavior in every magnetic field plane. In the xz plane main lobe magnitude is -37.7 dB A/m and for yz plane it is -30.3 dB A/m. At xy plane it can be seen that the side lobe level is -11.9 dB with angular width (3dB) 71.5$^\circ$.

The proposed prototype has been observed to provide radiation in all directions, matching the acceptable behaviour shown in simulations. Nonetheless, a minor difference is investigated at between the simulated and measured results in both planes as a result of constraints of the measurement setup and flaws in the 3D Yagi antenna.

**Figure 13:**
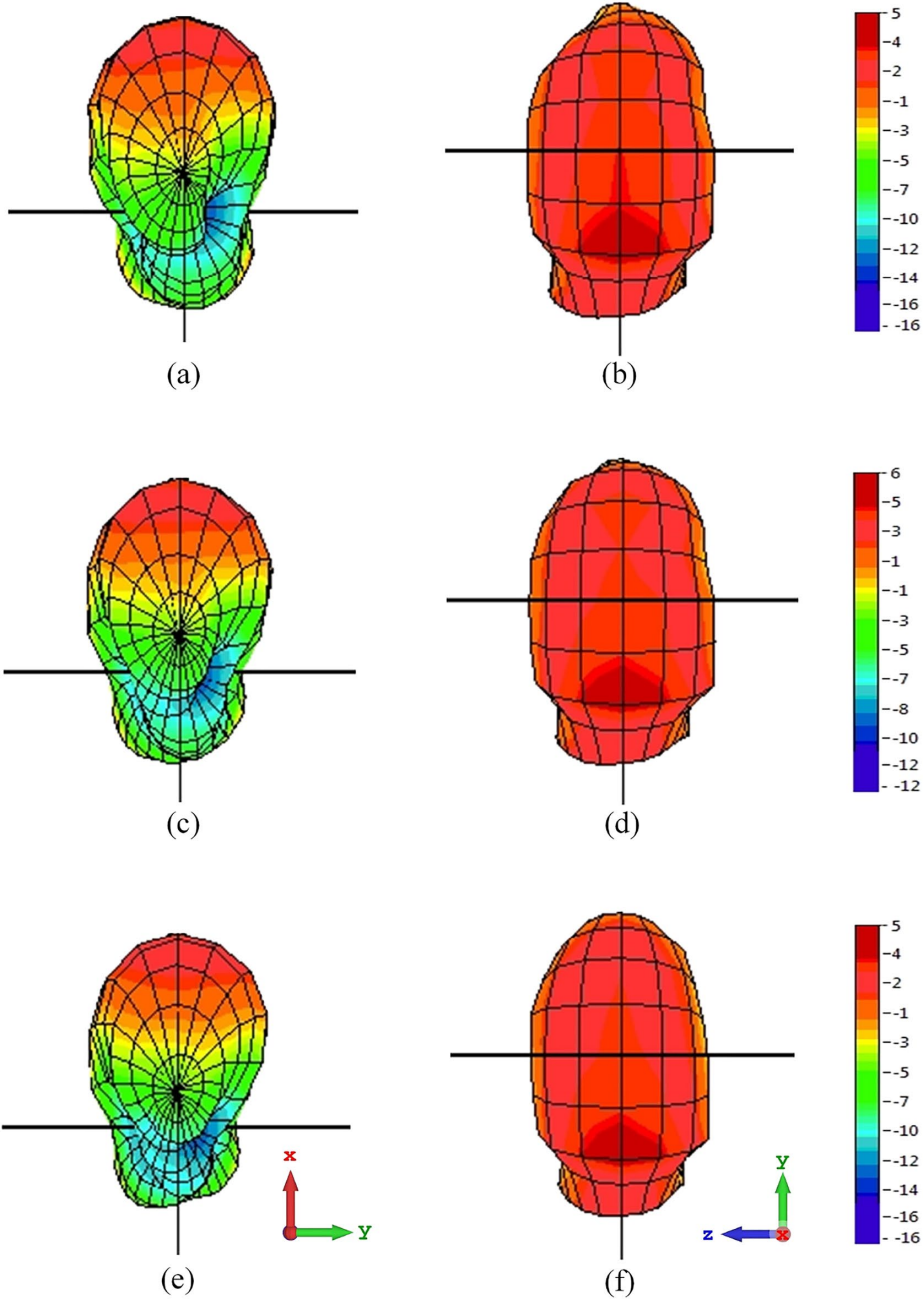
Measured 3D radiation pattern for (**a**) 3.4 GHz bottom view, (**b**) 3.4 GHz top view, (**c**) 3.5 GHz bottom view, (**d**) 3.5 GHz top view, (**e**) 3.6 GHz bottom view and (**f**) 3.6 GHz top view.

#### Radiation pattern (3D)

Anechoic chambers are used to measure 3D radiation patterns. Measure the antenna’s field from different angles with a probe or horn antenna. The antenna’s radiation pattern is plotted in 3D using this data. The pattern of radiation in a three-dimensional spherical coordinate system is depicted in Fig. 13. The projected measured 3D radiation pattern bottom view and front view at 3.4 GHz is shown in Fig. 13a and b. Figure 13c and d depict the 3D radiation pattern of the proposed measured Yagi antenna at 3.5 GHz. Finally, the measured 3D radiation pattern for 3.6 GHz is presented in Fig. 13e and f.

## Equivalent circuit modeling and simulation

Circuit design tools like Agilent ADS software and CST Studio simulation are used to create the antenna’s equivalent circuit, which is produced by the antenna’s impedance analysis. Maximum power transfer (at least 90%) from the input port to the antenna structure and radiation into free space is guaranteed by a return level of less than $-10$ dB at the resonance frequency. In order to transfer as much power as possible, it is necessary to match the antenna circuit’s impedance to the characteristic impedance of 50$\Omega$^40^. According to the principle of maximum power transmission, for a network to be considered “matched,” the load impedance and the input resistance (Z$_{load}$ = R$_{in}$) should be as close to equal as possible.

**Figure 14:**
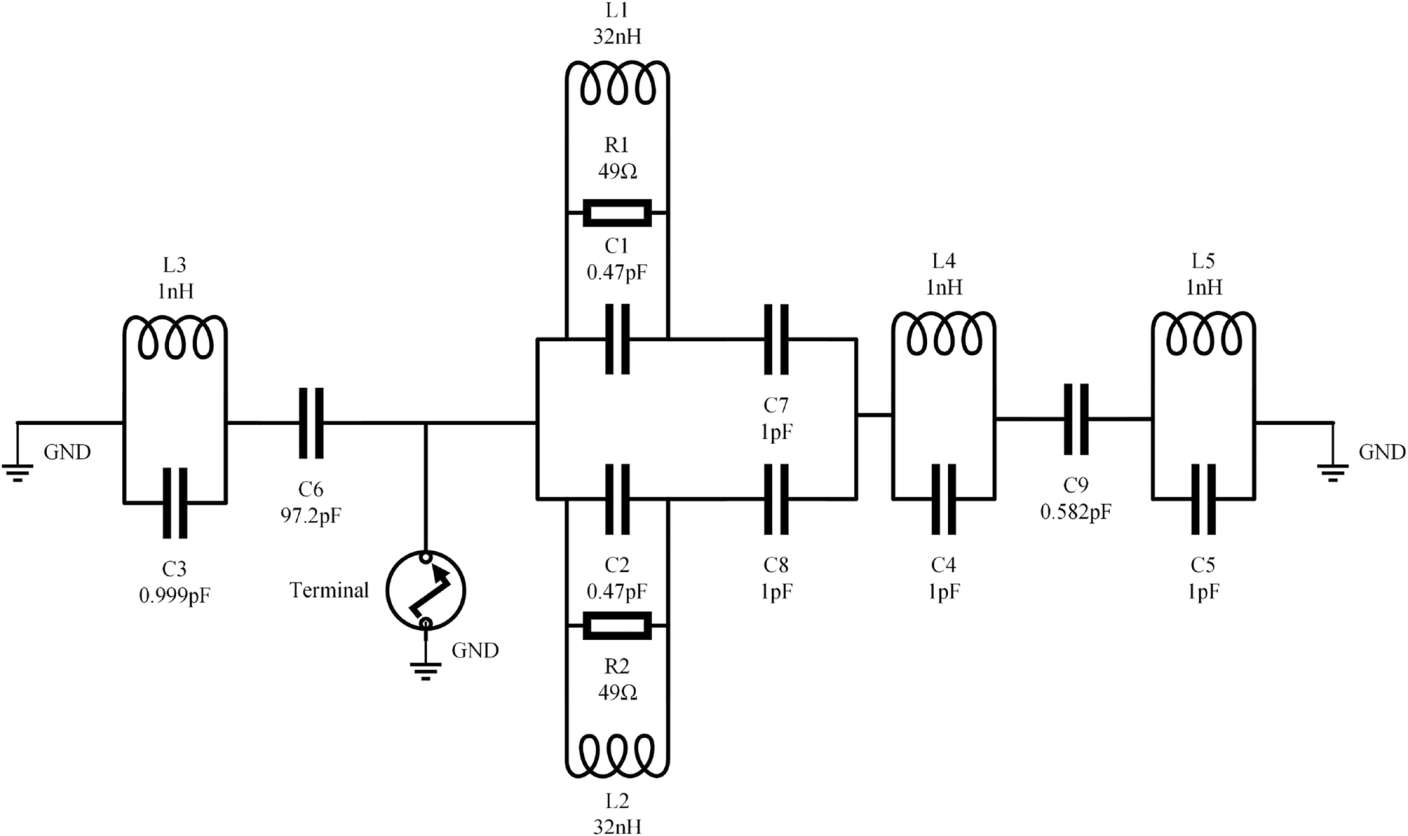
Equivalent circuit model of proposed antenna.

**Figure 15:**
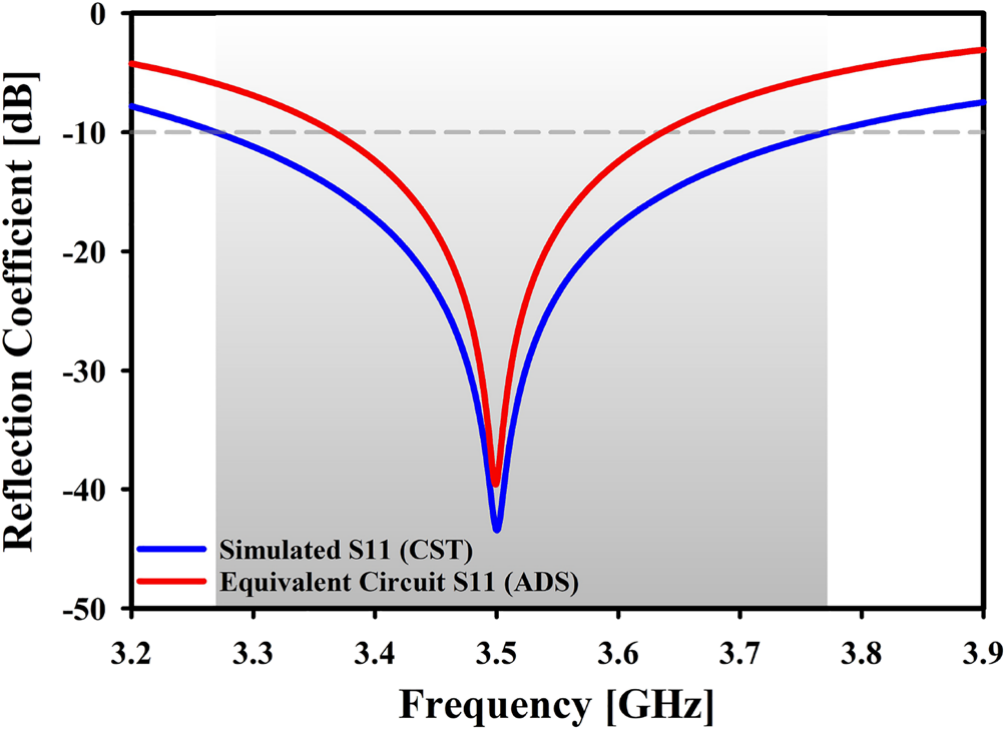
Simulated reflection coefficient of equivalent circuit in ADS and CST.

**Figure 16:**
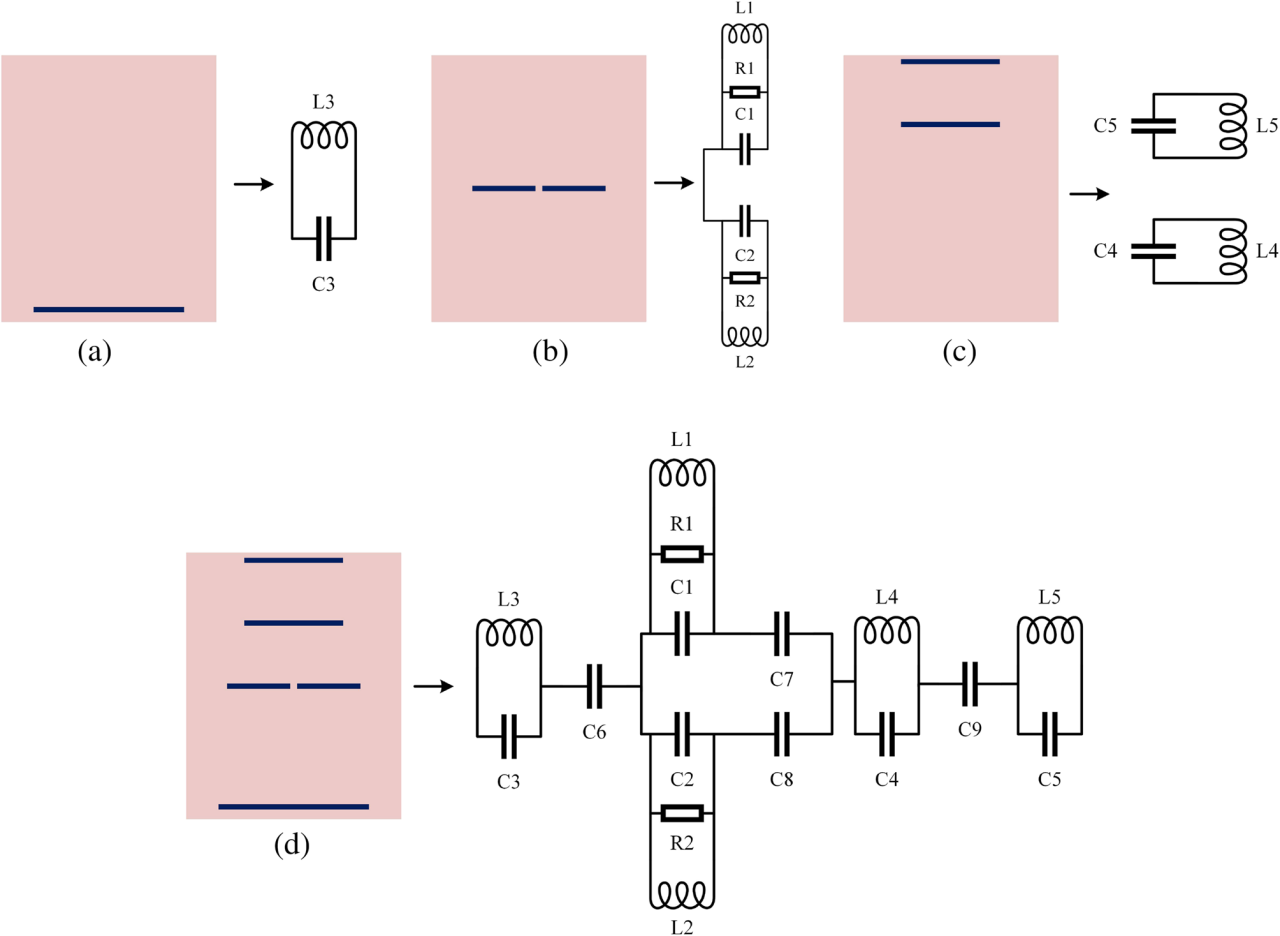
The development of the Yagi antenna’s equivalent circuit: (**a**) the reflector circuit model, (**b**) the dipole element circuit model, (**c**) the directors circuit model, (**d**) the final equivalent circuit model.

Finding a lumped element model (RLC circuit) with characteristics close enough to the proposed Yagi antenna is the basis of this method. After disassembling the antenna and proposing a similar circuit for each part, the final product is reassembled as Fig. 16a–d^41,42^. The final phase involves simulating the proposed antenna’s equivalent circuit model across its full frequency range using the R–L–C parameters. This model accurately represents the intended Yagi antenna operation. Clearly displayed the qualities of the notches in Fig. 14. The suggested Yagi antenna’s behavior is captured by this model rather accurately. The findings of the CST simulation are compared to the results of a similar circuit simulation using the S11 parameters in Fig. 15.

### Equivalent circuits of Yagi antenna dipole element

As part of the equivalent circuit, the proposed Yagi antenna was developed using transmission lines. Consequently, right dipole element of the antenna reproduces a parallel R1, L1, C1 circuit, left dipole element of the proposed antenna produces a parallel R2, L2, C2. Whereas C6 represents the gap between dipole and reflector as shown in Fig. 16b.

### Equivalent circuits of reflector and director

Reflector of the antenna produces a parallel of L3 and C3. Whereas C7 and C8 represents the gap between dipole and director1.The combination C4 and L4 symbolizes the first director, C5 and L5 denotes the second director as depicted in Fig. 16 a and 16c .The gap between director 2 and director 1 is signified by the letter C9.

## Machine learning methodology

ML approaches have seen much research and use in antenna designs during the past decade, due to their capacity to learn from observed or simulated antenna data through a training process. In ML-assisted optimization (MLAO), a computationally efficient model is built using ML techniques to predict the designated characteristics at the possible points in the design space using the training set generated at the sampled points based on the original computationally expensive model. Gaussian process regression (GPR), support vector machine (SVM), and artificial neural networks are just a few of the ML techniques included in MLAO approaches to antenna design^43^. To provide a high-level overview, machine learning may be defined as the extraction of useful information from data through the development of accurate prediction algorithms^44^. These algorithms have the potential to be useful in optimization, but their efficacy is contingent on the quality and quantity of the data that is gathered. Because of this, statistical analysis and machine learning are often considered to be synonymous terms. Regression methods are useful for expediting the optimization process since their ML assessment is much quicker than the numerical solution of a physical simulation model^45^. Regression models also help isolate the role of each design element in producing the desired results.

The methodology is composed of two separate sections. In the initial step of the process, the simulation software known as CST is used to build the antenna to operate at a frequency in the middle of the 5G spectrum and to extract the dataset produced through a parametric sweep.The next step is to train the dataset to apply machine learning models and to forecast which model will work best.

**Figure 17:**
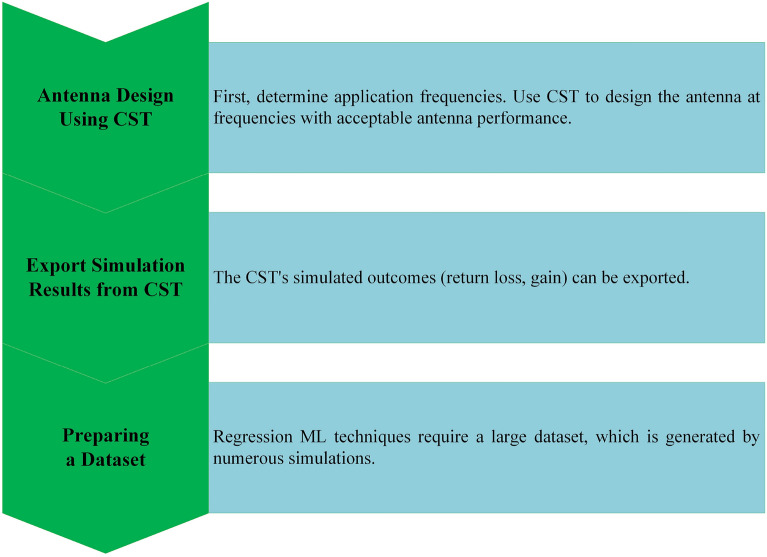
Data acquisition workflow for machine learning.

The methodology that is displayed in Fig. 17 will now be discussed in further detail. In the beginning determine the frequency of the middle band of the 5G application, which is 3.5 GHz. Utilize CST to design the antenna at frequencies where the performance of the antenna is satisfactory. With the use of a parametric sweep, it is possible to export the simulated parameters of CST, such as the length of the director, the size of the dipole, and the length of the ground and the reflector. Larger datasets can be helpful for regression machine learning algorithms in some cases, although this is not always the case. Several factors, including the problem’s complexity, the dimensionality of the input characteristics, and the model’s complexity, influence how much a larger dataset affects a regression model. In the end, 141 data samples are collected via the simulation with the aid of CST MWS, and a variety of regression machine learning (ML) methods are utilized to predict the gain and resonant frequency of the suggested Yagi antenna.

**Figure 18:**
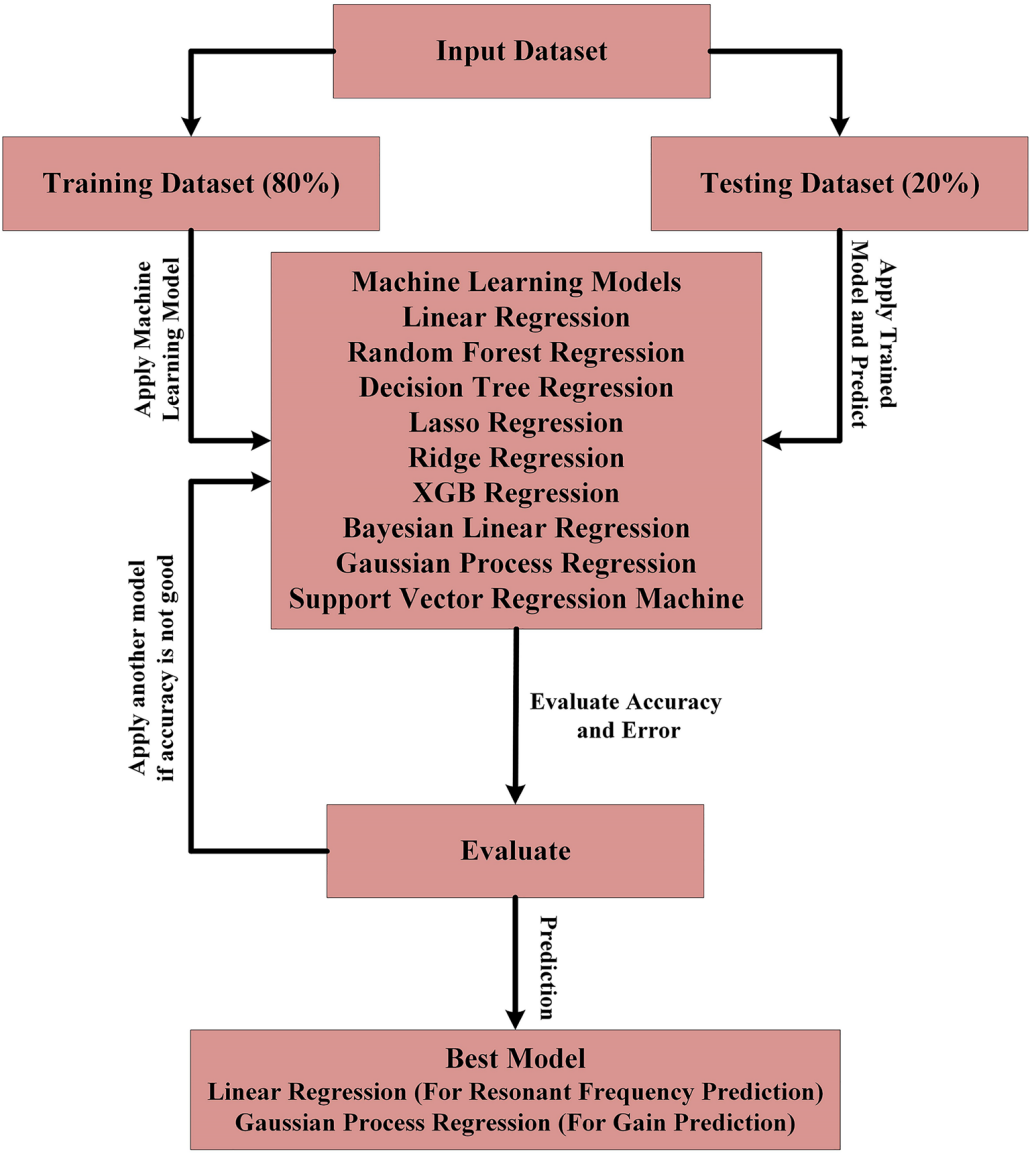
Flowchart illustrating the implementation of a machine learning algorithm.

The present study employs nine distinct machine-learning algorithms to generate predictions. The regression models under consideration include Linear Regression, Random Forest Regression, Decision Tree Regression, Lasso Regression, Ridge Regression, XGB Regression, Bayesian Linear Regression, Gaussian Process Regression, and Support Vector Regression Machine. These algorithms are selected based on their ability to perform regression analysis on non-linear datasets. Regression is the most suitable approach for implementing predictions, as the intended outcome is numerical values. A primary statistic in regression analysis, an error is so named due to its ubiquity. The flowchart depicted in Fig. 18 illustrates the development process of a machine learning algorithm. Upon analyzing the dataset, it was partitioned into two distinct segments that were obtained through a parametric sweep conducted on the CST simulation software.

All the machine learning study was conducted on Google’s simulated Python environment, termed google colab. To efficiently construct the Regression models, we used the sci-kit learn machine learning framework. Matplotlib was used for every analysis and visualization, but notably in the conclusion. The dataset can be divided into training and testing subgroups using the train-test split method. In this method, the data is split at random into two categories: training the model and testing its accuracy on new data. The following is an example from our linear regression technique showing how we use the scikit-learn module in Python to partition our data:
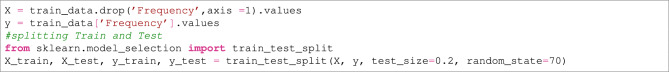


In the above bit of code, X stands for the feature matrix (the variables that serve as inputs), and y indicates the target variable (the variable whose value we wish to predict). When we specify that the test size should be 0.2, we set aside 20% of the data for testing while devoting the balance, or 80%, of the data to the process of model training. By fixing the random seed, the random state parameter guarantees reproducibility. We need a certain split between training and testing; therefore, we adjust the test_size option accordingly. After we have partitioned the data, we can use X_train and y_train to train our regression model, and then we can use X_test and y_test to evaluate the model’s performance on data for which it has not been trained.

As per the suggestion made in^46^, the first part of the study involved selecting 80% of the total dataset for training purposes, while the remaining 20% was reserved for testing in the second part. Subsequently, the training dataset is subjected to a machine-learning algorithm incorporating various features and labels. Upon completion of model training and cross-validation, the model can be effectively utilized to forecast the resonant frequency and realized gain for the intended inputs. Machine learning (ML) enables faster and more accurate predictions than results obtained through computer simulation technology (CST). As per the forecast, the optimal model for resonant frequency is Linear Regression, whereas, for realized gain, it is Gaussian Process Regression.

## Machine learning model selection

**Figure 19:**
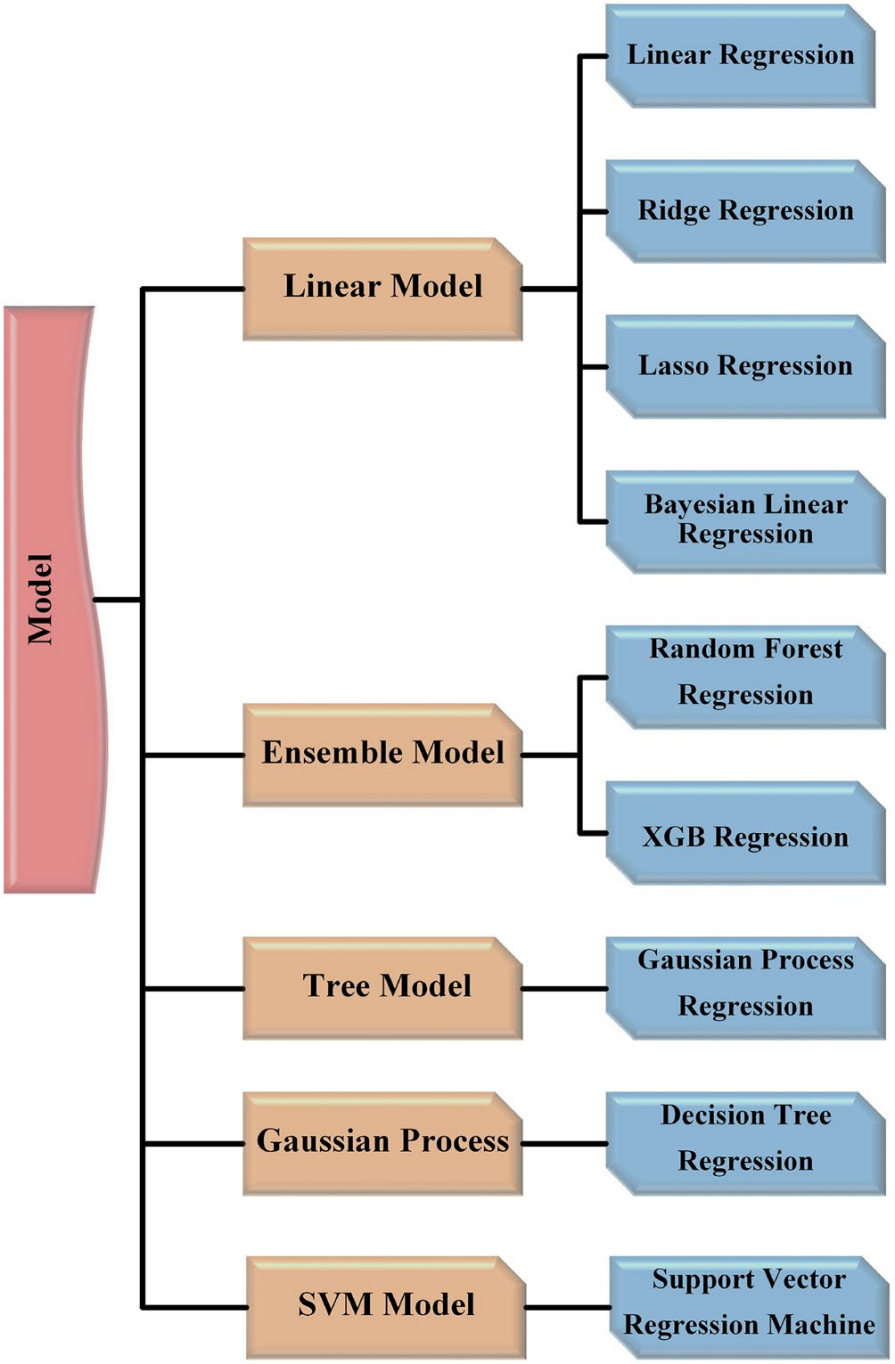
Splitting of regression algorithms.

The availability of a diverse range of models is of great value in attaining outstanding results. Regression evaluation, a statistical method, can be utilized to assess the connections among variables.^47^ Regression analysis is employed due to its ability to effectively address the issue at hand. Nine machine learning regression models are utilized that were deemed most effective, as illustrated in Fig. 19. The subsequent text provides a brief understanding of each of these.

**Table 2:** The resonant frequency prediction performance.

Algorithms	MAE (%)	MSE (%)	RMSE (%)	MSLE (%)	RMSLE (%)	MAPE (%)	R-Square (%)	Var Score (%)
Linear Regression	0.3177	0.0014	0.3802	0.0001	0.0842	0.0904	99.7976	99.8975
Random Forest Regression	0.3191	0.0023	0.4752	0.0001	0.1050	0.0907	99.6838	99.6921
Decision Tree Regression	0.4279	0.0028	0.5251	0.0001	0.1151	0.1206	99.6139	99.6148
Lasso Regression	5.3217	0.5772	7.5973	0.0277	1.6644	1.4952	19.1839	19.1984
Ridge Regression	0.3250	0.0015	0.3827	0.0001	0.0847	0.0923	99.7949	99.8918
XGB Regression	0.4315	0.0026	0.5056	0.0001	0.1112	0.1219	99.6421	99.6421
Bayesian Linear Regression	0.3451	0.0016	0.3990	0.0001	0.0882	0.0980	99.7771	99.8720
Gaussian Process Regression	0.3172	0.0015	0.3887	0.0001	0.0862	0.0903	99.7885	99.8909
Support Vector Regression Machine	0.7815	0.0079	0.8865	0.0004	0.1948	0.2211	98.8997	98.9471

**Linear regression** Linear regression, as described in reference^48^, establishes a linear relationship between the independent and dependent variables. Therefore, independent variables exhibit a corresponding alteration with respect to dependent factors. One vital assumption is that errors, which refer to the differences between anticipated and observed values, follow a normal distribution and exhibit uniform variance.

**Random forest regression** The process of classification and regression using random forests entails creating a group of tree forecasts. Each tree forecaster is constructed using an unknown vector that is selected autonomously of the input vector. The regression with tree forecasting method involves the substitution of class labels with values in numbers. The random forest regression algorithm builds a decision tree by utilizing variables at each node, as stated in reference^49^.

**Decision tree regression** According to literature^50^, regression trees are utilized for the prediction of constant target variables, such as numerical values. Supervised machine learning utilizing decision tree regression is a method for predicting constant target variables. This is a variant of the decision tree method that is used for tasks such as classification.

**Lasso regression** The Lasso regression technique is a form of linear regression which utilizes a reduction approach. Lasso regressions are frequently employed by researchers in modelling environments that involve numerous characteristics^51^, owing to their effectiveness in performing attribute selection.

**Ridge regression** Ridge regression is a useful technique when dealing with a substantial number of variables and aiming to minimize the coefficients of less important characteristics to zero. In the field of antenna architecture, various input attributes are taken into account, some of that may not have a significant impact on the outcome^52^.

**XGB regression** XGBoost’s internal optimizations accelerate the training process when dealing with large datasets. The software provides advanced functionalities such as regularization, parallel processing, and handling of incomplete data. Antenna developers have the ability to utilize either simulated or observed data in order to forecast antenna characteristics such as directivity, gain, and distributions of radiation through the assistance of XGBoost^53^.

**Bayesian linear regression** The Bayesian approach to linear regression involves the estimation of prior probabilities for the model variables, as opposed to the determination of the ideal value for stated variables^54^. An advantage of employing Bayesian Linear Regression lies in the ability to utilize the distribution that follows for the purpose of measuring the level of ambiguity in the forecasts made by the model. The utilization of probabilistic comprehension in the interpretation of forecasts can yield advantageous outcomes.

**Gaussian process regression** Gaussian process regression, often known as GPR, is a type of supervised machine learning approach that can be applied to activities including regression as well as classification. The use of ground-penetrating radar (GPR) has a number of advantages, including the fact that it can produce satisfactory findings even when working with a restricted collection of data and that it can provide measures of ambiguity for predictions^55^.

Support vector regression machineSupport Vector Regression Machine is a machine learning technique that is utilized for the purpose of regression analysis. This methodology utilizes the principles of Support Vector Machines (SVM) in order to make predictions of continuous numerical values. Support Vector Regression Machine (SVRM) aims to identify an optimal hyperplane by minimizing the number of margin violations. It achieves this by incorporating kernel functions to account for non-linear relationships. Support Vector Regression Machine (SVRM) is utilized in various domains, encompassing finance, time series analysis, and regression tasks requiring accurate numerical predictions^56^.

### Eight independent statistics

The mean absolute error (MAE), the mean squared error (MSE), the root mean square error (RMSE), the root mean squared logarithmic error (RMSLE), the mean percentage error (MPE), the mean absolute percentage error (MAPE), the coefficient of determination (R2), and the variance score-were used to evaluate the accuracy of the predictions. Mean absolute error (MAE) figures out the average difference between the values that were calculated and the values that were found. Equation (9) depicts the MAE^57^ formulation.

9 $$\begin{aligned} M A E=\frac{1}{n} \sum _{i=1}^n\left| y_i-{\hat{y}}_i\right| \end{aligned}$$

Where $\textrm{n}=$ number of errors $\left| y_i-{\hat{y}}_i\right| =$ error absolute

The mean squared error (MSE), is the type of regression loss function that is utilized most. The loss is the mean overseen data of the squared differences between true and predicted values. The MSE^58^ formulation is shown in Eq. (10).

10 $$\begin{aligned} M S E=\frac{1}{n} \sum _{i=1}^n\left( y_i-{\hat{y}}_i\right) ^2 \end{aligned}$$

Root mean squared error (RMSE) restores the unit to its original value by taking the Root of MSE. Equation (11) illustrates RMSE^59^ expression.

11 $$\begin{aligned} R M S E=\sqrt{\frac{1}{n} \sum _{i=1}^n\left( y_i-{\hat{y}}_i\right) ^2} \end{aligned}$$

The mean squared logarithmic error (MSLE) can be regarded as a ratio of the true and predicted values. MSLE^60^ equation is shown in Eq. (12).

12 $$\begin{aligned} M S L E=\frac{1}{n} \sum _{i=1}^n\left( \log \left( y_a\right) -\log \left( y_p\right) \right) ^2 \end{aligned}$$

Root Mean Squared Logarithmic Error (RMSLE) restores the unit to its original value by taking the Root of MSLE. The equation of RMSLE^61^ is shown in Eq. (13).

13 $$\begin{aligned} R M S L E=\sqrt{\left( \log \left( y_i+1\right) -\log \left( {\hat{y}}_i+1\right) \right) ^2} \end{aligned}$$

The mean absolute percentage error (MAPE) can be computed by first determining the difference between the actual value and the predicted value, and then dividing it by the actual value. Equation (14) depicts the MAPE^62^ formula.

14 $$\begin{aligned} M A P E=\frac{100 \%}{n} \sum \left| \frac{y_i-{\hat{y}}_i}{y_i}\right| \end{aligned}$$

The R-squared value indicates the accuracy of your model fit. When R$^2$ is close to 1, it indicates that the model provides a good fit for the data, whereas when it’s closer to 0, it indicates that the model isn’t all that good. When a model predicts an absurd outcome, R-squared can be negative. R-squared^63^ is expressed in Eq. (15).

15 $$\begin{aligned} R^2=1-\frac{\sum _{i=1}^N\left( y_i-{\hat{y}}_i\right) ^2}{\sum _{i=1}^N\left( y_i-{\bar{y}}_i\right) ^2} \end{aligned}$$

The explained variance score^64^ describes the error dispersion in each dataset. It is defined as in Eq. (16).

16 $$\begin{aligned} \text { explained varience }(y, {\hat{y}})=1-\frac{{\text {Var}}(y-{\hat{y}})}{{\text {Var}}(y)} \end{aligned}$$

## Result analysis M/L

**Figure 20:**
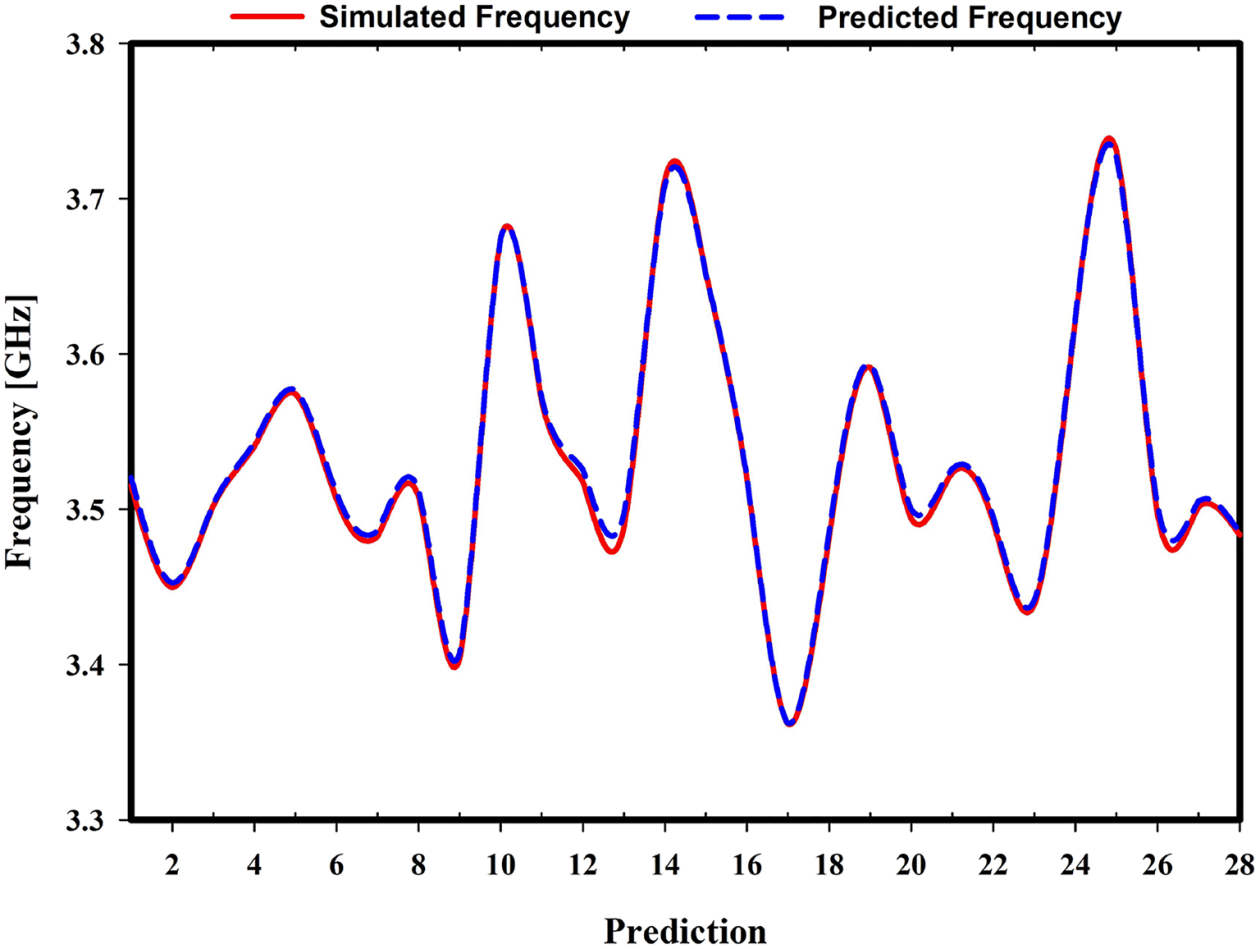
Simulated vs. predicted frequency using Linear Regression.

Table 2 compares the nine regression models’ abilities to predict resonant frequency using eight different parameters. The mean absolute error (MAE) and the mean absolute percentage error (MAPE) are both lowest when using the Gaussian Process Regression method, coming in at 0.3172% and 0.0903%, respectively. The mean squared error (MSE), root mean squared error (RMSE), and root mean squared absolute error (RMSLE) values for Linear Regression are 0.0014%, 0.3802%, and 0.0842%, respectively. When it comes to R-squared and variance score, Linear Regression has the highest accuracy at 99.7976% and 99.8975%, respectively. The fluctuation of the simulated and predicted frequency difference was depicted by the graph in Fig. 20 using Linear Regression. In the investigation, we tune between 3.35 and 3.75 GHz. We have 28 test observations. Table 3 shows the expected and simulated resonance frequencies and their values. Observations 12 and 15 had the highest and lowest discrepancies between simulated and predicted values, 0.0077 and 0.0001. Based on this data, LR is chosen because it predicts frequency more accurately than other ML models.

**Figure 21:**
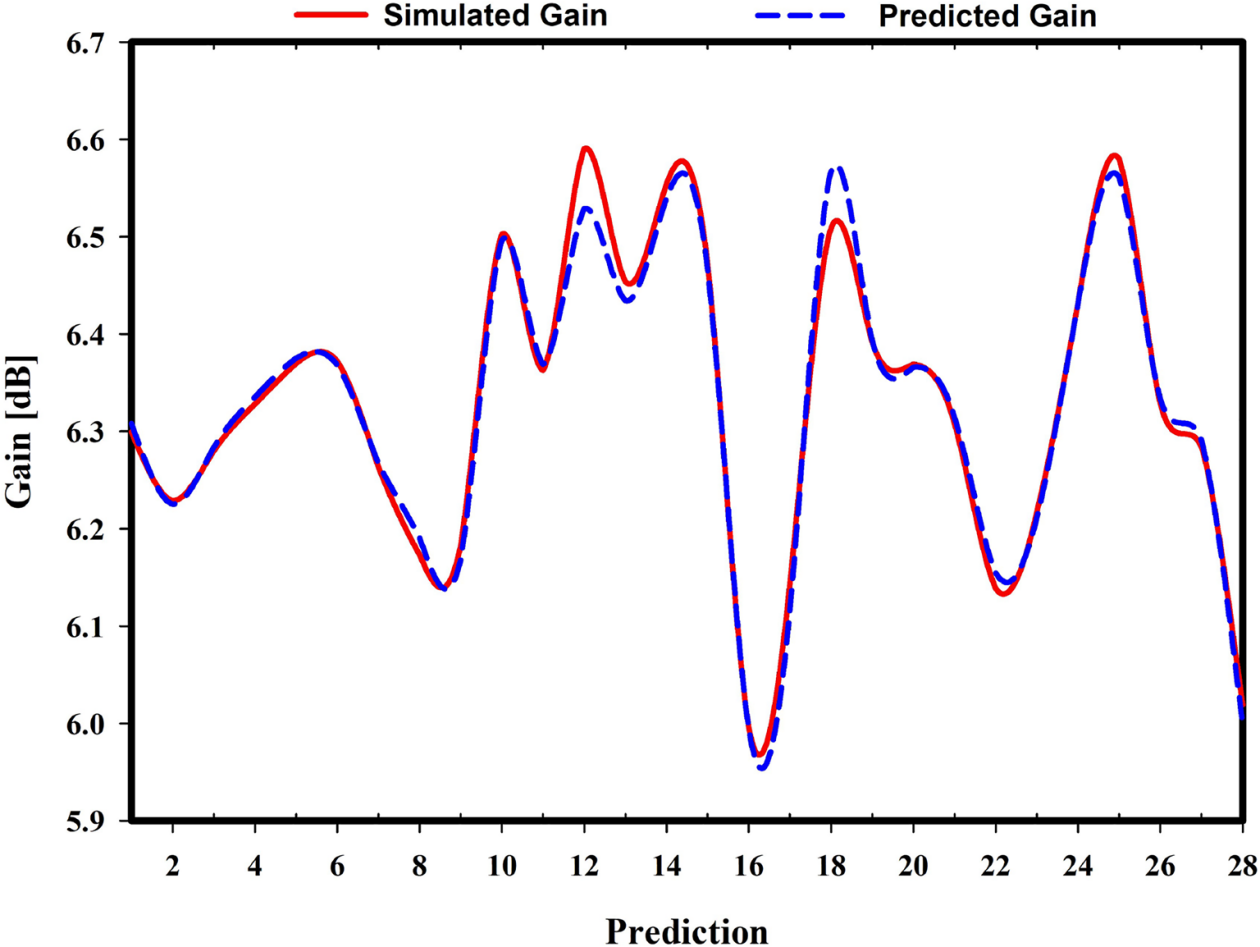
Simulated vs. predicted gain using Linear Regression Gaussian Process Regression (GPR).

Gain prediction accuracy for the nine regression models is compared in Table 4. Furthermore, eight distinct criteria were used to make this comparison. The MSE, MSLE, and MAPE values of 0.0375%, 0.0007%, and 0.1978%, respectively, are quite close to those found in both Gaussian Process Regression and Linear Regression, respectively. However, Gaussian Process Regression has the lowest error in terms of MAE and RMSE, and has the best accuracy for R-squared and variance score, with 98.4022% and 98.4200%, respectively. The graph in Fig. 21 depicts the volatility of the simulated and predicted gain difference using Gaussian Process Regression. Table 5 compares predicted and simulated gain. Observations 12 and 16 had the highest and lowest variances, 0.0617 and 0.0004, respectively. GPR was chosen because it predicts gain better than other ML models.

**Table 3:** Simulated and predicted resonant frequency comparison on Test set using Linear Regression.

No.	Simulated frequency (GHz)	Predicted frequency (GHz)	Difference (GHz)	No.	Simulated frequency (GHz)	Predicted frequency (GHz)	Difference (GHz)
1	3.5151	3.5203	0.0052	15	3.6516	3.6517	0.0001
2	3.4498	3.4526	0.0028	16	3.5195	3.521	0.0015
3	3.5024	3.5035	0.0011	17	3.3617	3.3623	0.0006
4	3.5404	3.543	0.0026	18	3.4815	3.4844	0.0029
5	3.5744	3.5768	0.0024	19	3.5913	3.5934	0.0021
6	3.5066	3.5105	0.0039	20	3.494	3.5003	0.0063
7	3.4827	3.4864	0.0037	21	3.5234	3.526	0.0026
8	3.508	3.5121	0.0041	22	3.4915	3.4952	0.0037
9	3.4037	3.4074	0.0037	23	3.4388	3.4413	0.0025
10	3.6751	3.6749	0.0002	24	3.6222	3.6225	0.0003
11	3.5689	3.5712	0.0023	25	3.7307	3.7271	0.0036
12	3.5178	3.5255	0.0077	26	3.4967	3.5022	0.0055
13	3.4884	3.4974	0.009	27	3.5017	3.5053	0.0036
14	3.713	3.7097	0.0033	28	3.4835	3.4853	0.0018

**Table 4:** The gain prediction performance.

Algorithms	MAE (%)	MSE (%)	RMSE (%)	MSLE (%)	RMSLE (%)	MAPE (%)	R-Square (%)	Var Score (%)
Linear Regression	1.2613	0.0375	1.9370	0.0007	0.2599	0.1978	98.4001	98.4177
Random Forest Regression	2.9294	0.6479	8.0491	0.0118	1.0872	0.4522	72.3752	74.7430
Decision Tree Regression	5.5339	2.2167	14.8887	0.0418	2.0449	0.8533	5.4812	14.7092
Lasso Regression	8.8406	2.0092	14.1748	0.0372	1.9288	1.3811	14.3286	18.2315
Ridge Regression	1.2658	0.0377	1.9420	0.0007	0.2605	0.1985	98.3920	98.4105
XGB Regression	2.6690	0.3121	5.5870	0.0056	0.7507	0.4148	86.6906	87.4399
Bayesian Linear Regression	1.2632	0.0376	1.9393	0.0007	0.2602	0.1981	98.3964	98.4144
Gaussian Process Regression	1.2610	0.0375	1.9358	0.0007	0.2598	0.1978	98.4022	98.4200
Support Vector Regression Machine	3.4624	0.4739	6.8843	0.0085	0.9239	0.5339	79.7920	81.9157

**Table 5:** Simulated versus predicted gain using linear regression gaussian process regression (GPR).

No.	Simulated gain (GHz)	Predicted gain (GHz)	Difference (GHz)	No.	Simulated gain (GHz)	Predicted gain (GHz)	Difference (GHz)
1	6.2993	6.3079	0.0087	15	6.472	6.4676	0.0044
2	6.229	6.2253	0.0037	16	5.9963	5.9959	0.0004
3	6.2798	6.2835	0.0037	17	6.1429	6.1142	0.0287
4	6.3281	6.3352	0.0071	18	6.5091	6.5668	0.0577
5	6.3698	6.3753	0.0055	19	6.3916	6.394	0.0024
6	6.3718	6.3679	0.0039	20	6.3688	6.3659	0.0029
7	6.2631	6.2667	0.0037	21	6.3078	6.3148	0.007
8	6.1731	6.1902	0.0171	22	6.1387	6.1551	0.0164
9	6.1841	6.1698	0.0143	23	6.2177	6.2114	0.0063
10	6.5027	6.4966	0.0061	24	6.4323	6.4304	0.0019
11	6.3627	6.3688	0.006	25	6.5796	6.561	0.0186
12	6.5907	6.529	0.0617	26	6.3282	6.3336	0.0053
13	6.4537	6.4344	0.0192	27	6.2839	6.2912	0.0073
14	6.5544	6.5397	0.0147	28	6.0192	6.0008	0.0184

In^22^, the authors utilized machine learning regression models to forecast the Return loss level. The Mean Squared Error (MSE) has been computed for error estimation, while the R2 Score has been exclusively utilized for accuracy evaluation. The crucial variance score for accuracy prediction is disregarded by them. The Mean Squared Error (MSE) and R-squared (R2) metrics are computed in the context of Random Forest Regression, Decision Tree Regression, and XGB Regression. The error rate exhibits a significant elevation across all models. The percentage exceeds 50%. The table displays numerical values. Resonant frequency prediction was conducted in a previous research study^65^ utilizing Random Forest Regression, Decision Tree Regression, and XGB Regression. The R-squared values for all models are greater than 97%, and the Random Forest Regression exhibits an error rate of 32%. The Decision Tree Regression model yielded a percentage score of 51%, while the XGB Regression model produced a score of 33%. The authors in^66^ employed six machine learning regression models to forecast the resonant frequency. The Variance score is utilized for precision evaluation while R2 is disregarded. The Linear Regression model exhibits an accuracy of approximately 76% and an error rate of 52.2%, while the Decision Tree Regression model demonstrates an accuracy of 99% and an error rate of 0.71%. In a previous study^67^, it was reported that the Decision Tree Regression Model exhibited an error rate of 11.33% and an accuracy rate of 67.5%. The prediction of return loss is performed in^68^. An accuracy rate of approximately 57.49% has been attained, while a significant error rate of approximately 62.2% is evident. This study employs several regression models, namely Linear Regression, Random Forest Regression, Decision Tree Regression, Ridge Regression, XGB Regression, Bayesian Linear Regression, and Gaussian Process Regression. The regression methods are utilized to predict both the resonant frequency and gain. The presented material exhibits a superior level of precision and a lower margin of error compared to other sources in all regards as discussed and presented in table 6.

**Table 6:** Machine learning performane comparison.

Ref	Model		Linear Regression	Random Forest Regression	Decision Tree Regression	Ridge Regression	XGB Regression	Bayesian Linear Regression	Gaussian Process Regression	Support Vector Regression Machine
Ref.^22^	Return Loss Prediction	$R^2$ Score	N/A	96.1%	91.9%	N/A	93.9%	N/A	N/A	N/A
		V-Score	N/A	N/A	N/A	N/A	N/A	N/A	N/A	N/A
		MSE	N/A	50.9%	93.3%	N/A	79.4%	N/A	N/A	N/A
	Gain Prediction	N/A								
Ref.^65^	Resonant Frequency Prediction	$R^2$ Score	N/A	99%	97%	N/A	98%	N/A	N/A	N/A
		V-Score	N/A	N/A	N/A	N/A	N/A	N/A	N/A	N/A
		MSE	N/A	32%	51%	N/A	33%	N/A	N/A	N/A
	Gain Prediction	N/A								
Ref.^66^	Resonant Frequency Prediction	$R^2$ Score	N/A	N/A	N/A	N/A	N/A	N/A	N/A	N/A
		V-Score	76%	98%	99%	76%	99%	N/A	N/A	N/A
		MSE	52.2%	3%	0.71%	51%	1%	N/A	N/A	N/A
	Gain Prediction	N/A								
Ref.^67^	Resonant Frequency Prediction	$R^2$ Score	N/A	N/A	N/A	N/A	N/A	N/A	N/A	N/A
		V-Score	N/A	79.9%	67.5%	N/A	83.9%	N/A	N/A	N/A
		MSE	N/A	6.88%	11.33%	N/A	5.56%	N/A	N/A	N/A
	Gain Prediction	N/A								
Ref.^68^	Resonant Frequency Prediction	$R^2$ Score	N/A	99.96%	N/A	N/A	97.52%	57.49%	N/A	N/A
		V-Score	N/A	N/A	N/A	N/A	N/A	N/A	N/A	N/A
		MSE	N/A	0.04%	N/A	N/A	3%	62.2%	N/A	N/A
	Gain Prediction	N/A								
Proposed	Resonant Frequency Prediction	$R^2$ Score	99.7976%	99.6838%	99.6139%	99.7949%	99.6421%	99.7771%	99.7885%	98.8997%
		V-Score	99.8975%	99.6921%	99.6148%	99.8918%	99.6421%	99.8720%	99.8909%	98.9471%
		MSE	0.0014%	0.0023%	0.0028%	0.0015%	0.0026%	0.0016%	0.0015%	0.0079%
	Gain Prediction	$R^2$ Score	98.4001%	72.3752%	5.4812%	98.3920%	86.6906%	98.3964%	98.4022%	79.7920%
		V-Score	98.4177%	74.7430%	14.7092%	98.4105%	87.4399%	98.4144%	98.4200%	81.9157%
		MSE	0.0375%	0.6479%	2.2167%	0.0377%	0.3121%	0.0376%	0.0375%	0.4739%

Table 7 presents a comparative analysis of the computational performance between the proposed approach and the model based on the CST EM Simulator according to the analysis done by the authors in^69–71^. The simulations were conducted utilizing the specified simulation setup. The system is equipped with an Intel(R) Core (TM) i3-8145U CPU operating at a frequency of 2.10 GHz. Additionally, it has a total of 12.0 GB of RAM installed. The provided information includes descriptions of a model consisting of a single unit element and a set of 8 regression models. Additionally, the total duration required to obtain optimized models using both the CST EM Simulator-based model and the proposed approach is provided.

The overall cost of the proposed approach can be determined through the utilization of total RAM and the duration of time. A total of 141 data samples were utilized to assess the performance of the regression models. The verification process utilizes a total of 28 samples, while the training process involves 113 data points. To obtain the output of regression models, Google Collab was utilized, resulting in an approximate memory consumption of 200 MB. The retrieval of output for each regression model was accomplished within a time frame of 0.1–0.2 s. In contrast, the time required to obtain output in CST EM Simulator Models and achieve the desired result is approximately 2 min 52 s for Single unit element CST EM Simulator, 10 min 31 s for Single unit element CST EM Simulator (Medium Complexity Mesh Configuration) and, 25 min 10 s for Single unit element CST EM Simulator (High Complexity Mesh Configuration). The proposed method demonstrates a significant increase in speed compared to the design approach based on CST EM Simulator Models. In particular, the observed time difference between the two methods is nearly 100-fold, with the first method taking approximately 25 min in high complexity mesh configuration and the second method requiring a mere 0.2 s. Performance evaluation of the suggested method and the CST EM Simulator based design with respect to the overall design process and simulation cost.

**Table 7:** Performance evaluation of the suggested method and the CST EM simulator based design with respect to the overall design process and simulation cost.

Model	Model configuration	Time
Single unit element CST EM Simulator	Cells per wavelength =15 Cells per max model box edges = 20 Fraction of maximum cell near to model =20 Mesh size = 146,832	2 minute 52 second
Single unit element CST EM Simulator (Medium Complexity Mesh Configuration)	Cells per wavelength =30 Cells per max model box edges = 20 Fraction of maximum cell near to model =20 Mesh size = 259,200	10 minute 31 second
Single unit element CST EM Simulator (High Complexity Mesh Configuration)	Cells per wavelength =45 Cells per max model box edges = 20 Fraction of maximum cell near to model =20 Mesh size = 657,096	25 minute 10 second
Single Run of 8 Regression Models (Proposed approach)	The regression Model is generated using 141 iterations based on Python by collecting data through CST EM simulator (Medium Complexity Mesh Configuration)	0.1-0.2 Second/Model

## Conclusion

The performance of the proposed antenna is evaluated in this study by utilising a variety of methodologies, including simulation, measurement, the development of an RLC equivalent circuit model, as well as machine learning strategies for prediction. The antenna works in the Sub-6 GHz (n78) band for 5G applications. It has a maximum gain of 6.57 dB, a directivity of 6.79 dBi, and an efficiency of 97%.In both the ADS and the CST simulations, it has been noticed that the bandwidth of the n78 band is practically identical to one another.The reflection coefficient, gain, efficiency and radiation pattern that were produced as a result of the simulation are pretty comparable to the ones that were produced as a consequence of the measurements. In addition, nine machine learning algorithms were designed to calculate the Yagi-Uda antenna’s resonance frequency and gain. In terms of predicting the resonant frequency, the predicted results show that the error performances of the Linear regression (LR) model are relatively better than other models. On the other hand, when it comes to predicting the gain, the Gaussian Process Regression (GPR) model shows better performance than other models. It has covered 14.77% of the bandwidth between 3.26 GHz and 3.78 GHz, making it a promising candidate for the n78 band in the 5G communication system.In light of the fact that the simulated and measured results correlate very well and that the constructed Yagi antenna provides complete coverage of all n78 frequency bands, it is possible to see this antenna as an ideal model for applications operating at sub-6 GHz frequencies.

## Data Availability

The datasets used and analyzed during the current study available from the corresponding author on reasonable request.
